# Digital Predistortion of Wideband Power Amplifiers Using Functionally Decoupled Envelope-Assisted Attention-Guided Recurrent Architecture

**DOI:** 10.3390/s26185945

**Published:** 2026-09-19

**Authors:** Bingwen Qiu, Xiaoyu Li, Yunjie Zhao

**Affiliations:** 1 College of Computer and Information Engineering, Xinjiang Agricultural University, Urumqi 830052, China; 220234627@stu.xjau.edu.cn (B.Q.);; 2 Engineering Research Center of Intelligent Agriculture, Ministry of Education, Urumqi 830052, China; 3 Xinjiang Agricultural Informatization Engineering Technology Research Center, Urumqi 830052, China

**Keywords:** digital predistortion, wideband power amplifier, neural predistortion, functional decomposition, recurrent neural network, complexity–performance trade-off

## Abstract

Wideband power amplifiers (PAs) operating with high-order modulation signals exhibit strong nonlinear distortion and dynamic memory effects, making real-time digital predistortion (DPD) increasingly challenging under strict computational constraints. This work proposes a functionally decoupled neural DPD architecture, termed EA-CCF-AttGRU, which explicitly separates instantaneous nonlinear feature representation from temporal memory compensation within a unified end-to-end framework. Instead of introducing envelope features, cross-channel fusion, and recurrent attention as isolated modules, the proposed architecture assigns different compensation functions to dedicated components: envelope-assisted augmentation and point-wise cross-channel fusion enhance instantaneous nonlinear representation, while attention-guided recurrent modeling captures dynamic memory effects. A global linear bypass further reduces the burden of nonlinear compensation by preserving the linear transformation. Experimental results under a 160 MHz 1024-ary quadrature amplitude modulation (1024-QAM) baseband excitation with a 10.38 dB peak-to-average power ratio (PAPR) demonstrate that the proposed method achieves an adjacent channel leakage ratio (ACLR) of −65.91 dBc, a normalized mean square error (NMSE) of −57.84 dB, and an error vector magnitude (EVM) of 0.07% with only 6009 trainable parameters. The proposed architecture achieves an effective complexity–performance trade-off for wideband DPD applications and provides potential for future hardware-oriented implementation and synthesis validation.

## 1. Introduction

Next-generation wireless communication systems increasingly employ wider channel bandwidths, carrier aggregation, and high-order modulation to improve spectral efficiency and data throughput. These signal formats generally exhibit high peak-to-average power ratios (PAPRs), forcing radio-frequency (RF) power amplifiers (PAs) to operate closer to their nonlinear region and thereby aggravating both in-band distortion and out-of-band spectral regrowth. As the signal bandwidth increases, PA distortion becomes increasingly dependent not only on the instantaneous input but also on its recent signal history, leading to pronounced dynamic memory effects. Digital predistortion (DPD) is therefore widely employed to compensate for nonlinear and memory-dependent distortion while enabling efficient PA operation.

Memory-aware behavioral modeling has long been central to PA linearization. Volterra-family formulations provide a systematic representation of nonlinear systems with memory, and recent fractional-polar Volterra formulations continue to extend this analytical framework for baseband PA modeling [1]. Earlier work explicitly demonstrated the importance of signal-history-dependent PA memory effects and developed structured behavioral models to represent them [2]. The generalized memory polynomial (GMP) subsequently provided a practical reduction in the Volterra representation by retaining dominant nonlinear memory interactions with substantially lower implementation complexity [3]. These developments established a fundamental trade-off that remains relevant to modern DPD: sufficiently expressive memory modeling is required for wideband linearization, whereas increasing model order and memory depth generally increases computational and implementation cost.

Data-driven approaches have progressively introduced neural networks to improve nonlinear representation and memory modeling. Real-valued time-delay neural networks (RVTDNNs) use delayed in-phase/quadrature samples to characterize nonlinear PA behavior with memory [4]. The augmented RVTDNN (ARVTDNN) further incorporates envelope-dependent terms into the real-valued input representation, enriching the nonlinear basis while maintaining a compact neural formulation [5]. Recurrent architectures subsequently introduced explicit sequential state modeling; for example, BiLSTM-based PA modeling associates PA memory behavior with recurrent sequence processing [6]. Augmented-LSTM and 1D-CNN-LSTM architectures further combine enriched input representations with recurrent processing, with the CNN-LSTM variant reducing the feature dimension presented to the recurrent stage to improve the complexity–performance balance [7]. Attention-assisted recurrent structures have also been explored, including GRU-Attention for millimeter-wave transmitter linearization [8] and LSTM-based DPD with additive attention [9], while residual temporal convolution has been employed for digital-PA predistortion [10]. These studies demonstrate that delayed neural modeling, envelope-derived augmentation, recurrent memory representation, temporal convolution, and attention-based weighting are established mechanisms in neural DPD.

Importantly, continued DPD research is not limited to incremental refinement of conventional narrowband or sub-6-GHz transmitters. Recent studies have extended neural and digital predistortion to emerging operating scenarios, including D-band PA linearization [11], contiguous carrier aggregation for sub-THz transmitters [12], millimeter-wave GaN PAs for integrated communication, sensing, and power-transfer applications [13], massive-MIMO transmitters [14], and MIMO systems affected by nonlinear interchannel coupling [15]. These developments show that PA linearization remains an active problem as transmitters move toward wider instantaneous bandwidths, higher carrier frequencies, denser antenna configurations, and more demanding spectral-efficiency requirements. Accordingly, the research focus has increasingly shifted from whether nonlinear compensation can be achieved to whether accurate compensation can be maintained under increasingly restrictive computational, memory, adaptation, and implementation budgets.

Several recent studies have therefore focused directly on reducing architectural or system-level overhead. Look-up-table-assisted BiLSTM predistortion assigns static and dynamic compensation functions to cascaded submodels [16], whereas simplified augmented real-valued time-delay neural networks reduce the complexity of envelope-assisted neural predistortion [17]. Adaptation under changing operating conditions has been investigated through linear-update neural DPD for previously unseen states [18] and joint power-back-off/DPD optimization [19]. Other approaches address practical system constraints, including neural-network-assisted DPD under sub-Nyquist sampling [20], safe deep-reinforcement-learning-based local and remote predistortion [21], phase-filtered neural DPD [22], and enhanced fully connected neural predistorters [23]. These studies illustrate that contemporary DPD design must account not only for nonlinear approximation accuracy but also for adaptation, feedback requirements, and computational cost.

Efficient temporal modeling and deployment-oriented optimization have likewise become important research directions. TCN-DPD employs temporal convolutional networks to achieve parameter-efficient wideband memory modeling [24], while cross-architecture knowledge distillation has been investigated to reduce the deployment burden of terahertz/mmWave predistortion models [25]. OpenDPD introduced an open-source end-to-end framework for reproducible PA modeling and DPD benchmarking [26], and OpenDPDv2 further developed a unified learning and optimization framework for neural DPD [27]. Specialized recurrent architectures such as APNRRU combine phase normalization, recurrent processing, and envelope-related states to obtain high linearization accuracy with modest model complexity [28]. Mixed-precision neural DPD explicitly reduces numerical implementation cost [29], whereas DeltaDPD exploits dynamic temporal sparsity in recurrent networks to reduce arithmetic activity and memory access [30]. Collectively, these developments indicate that modern neural DPD is increasingly evaluated through a joint consideration of linearization accuracy, parameter count, arithmetic workload, memory requirement, numerical precision, and implementation efficiency.

At the level of individual neural components, convolutional networks have already been established for behavioral modeling and predistortion of wideband PAs [31], while the long short-term memory mechanism itself provides the well-established gated recurrent foundation underlying many sequence-processing architectures [32]. Therefore, this work does not claim envelope augmentation, convolution, recurrent modeling, attention, residual processing, quantization, or other individual neural mechanisms as independently novel contributions.

Instead, the architectural question investigated in this work is how instantaneous nonlinear feature construction and temporal-memory evolution can be assigned to distinct lightweight functions within a unified end-to-end predistorter. In representative neural DPD architectures, temporal information is commonly introduced through delayed input samples, recurrent hidden states, temporal convolutions, or combinations of these mechanisms. In contrast, the present work explicitly constrains the front-end cross-channel fusion to operate only on features associated with the same timestamp and subsequently assigns temporal-memory evolution to the recurrent stage. Based on this design principle, an EA-CCF-AttGRU architecture is developed to investigate whether such functional organization can improve the complexity–performance trade-off. Established envelope-derived features are first incorporated as parameter-free amplitude-related augmentation. A kernel-size-one cross-channel fusion (CCF) stage then performs nonlinear interaction among feature channels at the current timestamp without explicit cross-timestamp mixing. Temporal dependencies are subsequently modeled by an attention-guided gated recurrent unit (AttGRU), while an independent global linear bypass represents the dominant affine compensation trajectory and reduces the mapping burden imposed on the nonlinear recurrent branch. The proposed architecture is therefore evaluated as a functionally organized DPD structure rather than as a collection of individually novel neural modules.

The main contributions of this work are as follows:

(1) Explicit functional role assignment within a unified neural DPD architecture. A single-box EA-CCF-AttGRU is developed in which established envelope-derived augmentation and strictly point-wise CCF are assigned to instantaneous nonlinear feature construction, whereas temporal-memory evolution is handled by the subsequent AttGRU. The CCF operates only across feature channels associated with the same timestamp and therefore introduces no explicit cross-timestamp dependency at the front-end fusion stage. A global linear bypass additionally represents the dominant affine compensation trajectory.
(2) Controlled evaluation of architectural benefit under comparable model capacity. The proposed architecture is evaluated through approximately iso-parameter benchmarking against representative recurrent and temporal-convolutional baselines, together with component-level ablation and architectural-variant studies. This evaluation is designed to determine whether the observed linearization improvement persists when differences in parameter count are substantially controlled, rather than attributing the performance gain to increased network size alone.
(3) Implementation-oriented complexity and robustness characterization. In addition to trainable parameter count, the proposed DPD is characterized in terms of dense arithmetic, element-wise and nonlinear operations, recurrent-state storage, temporal buffering, and numerical precision. Its behavior under input-power variation, feedback noise, fractional timing mismatch, block-wise adaptation, and post-training quantization is further evaluated. Under the 160 MHz 1024-QAM test condition, the selected 6009-parameter configuration achieves an ACLR of −65.91 dBc, an NMSE of −57.84 dB, and an EVM of 0.07%. These results characterize an observed algorithmic complexity–performance operating point without claiming synthesized FPGA/ASIC latency, resource utilization, or power consumption.

The remainder of this paper is organized as follows. Section 2 presents the wideband PA model and differentiable DPD formulation. Section 3 describes the functional organization and internal operations of the EA-CCF-AttGRU architecture. Section 4 presents the experimental setup, controlled benchmarking, linearization performance, ablation studies, complexity–performance analysis, robustness evaluation, adaptation analysis, and implementation-oriented discussion. Section 5 concludes the paper.

## 2. System Model and DPD Formulation

### 2.1. Wideband PA Nonlinearity and Digital Twin Modeling

Under wideband excitation, particularly with high-PAPR Orthogonal frequency division multiplexing (OFDM) signals, the PA exhibits prominent nonlinear distortion and dynamic memory effects. Let x(n)=I(n)+jQ(n) denote the original discrete-time complex baseband signal, comprising in-phase and quadrature components. To compensate for these distortions, a digital predistorter maps the original sequence to a predistorted signal z(n) which is then fed to the RF transmitter.

Under wideband operation, the normalized PA output y(n) at the current time index depends not only on the current predistorted input z(n) but also on a finite history of preceding inputs. A generalized discrete-time behavioral model of a PA with memory effects can therefore be expressed as follows:

(1) $$y(n)=f_{PA}(z(n),z(n-1),\ldots ,z(n-M))$$

where $f_{PA}(\cdot )$ denotes the complex nonlinear transfer function of the amplifier with dynamic memory effects, n is the discrete-time index, and M is the maximum memory depth. Capturing this high-dimensional nonlinear mapping requires an expressive behavioral model. Therefore, a high-fidelity digital twin of the wideband PA is employed as the differentiable forward environment for DPD optimization. Implemented with a deep GRU backbone, this surrogate achieves an NMSE of −38.45 dB and captures the dominant dynamic nonlinear behavior of the PA.

### 2.2. Differentiable Cascaded DPD Training with a Fixed PA Surrogate

**Figure 1:**
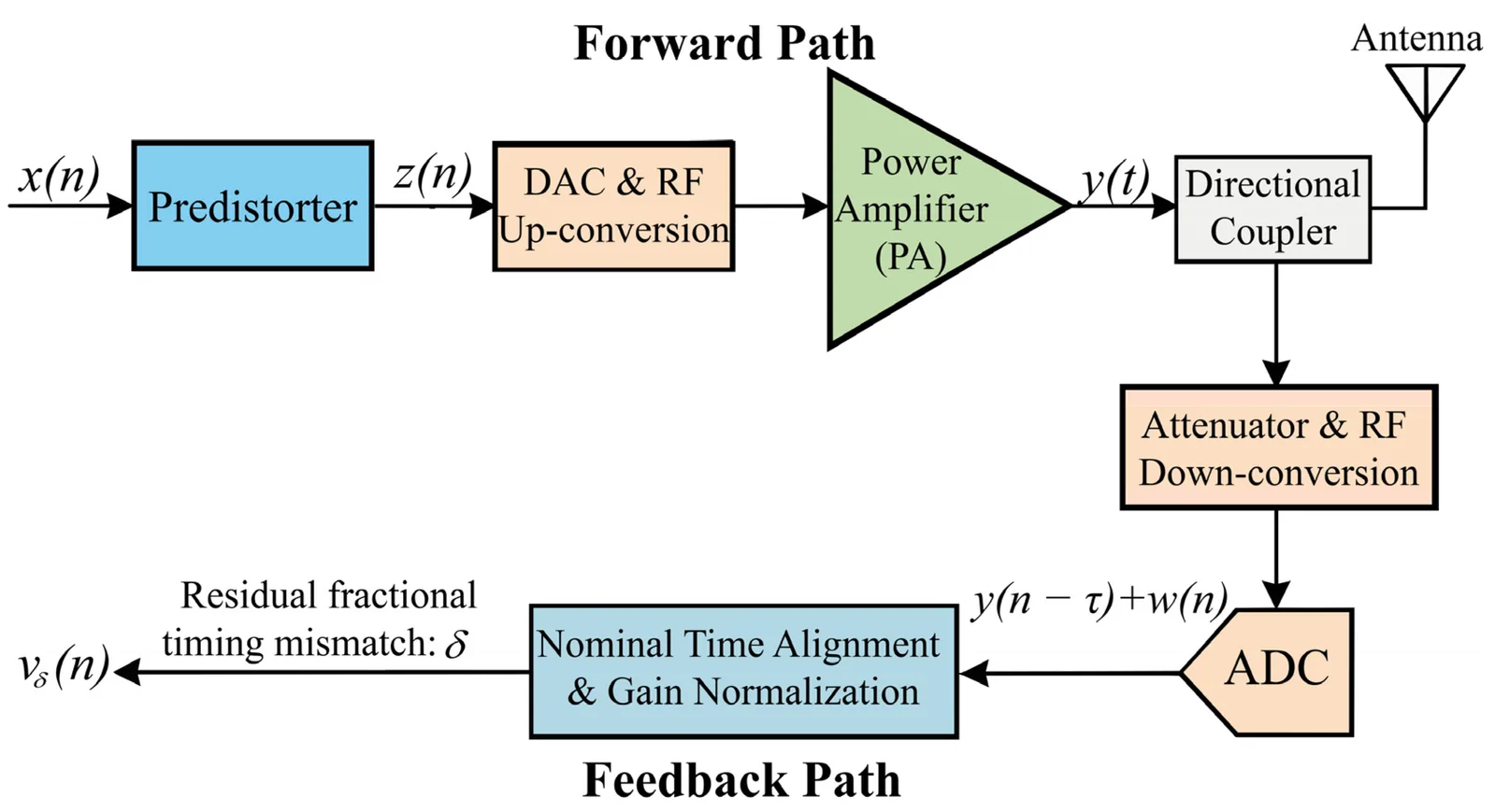
System-level DPD transmitter and feedback synchronization context. The arrows indicate the forward signal flow and feedback observation path. The colored blocks represent different functional modules: blue blocks denote digital signal processing and synchronization modules, orange blocks denote RF conversion and acquisition components, and the green block denotes the power amplifier. The PA output is observed through a directional coupler and a feedback receiver comprising attenuation, RF down-conversion, ADC sampling, nominal time alignment, and gain normalization. After nominal synchronization, the resulting feedback sequence is denoted by $v_{\delta }(n)$, where a residual fractional timing mismatch δ may remain.

Digital predistortion introduces a nonlinear inverse mapping before the PA so that the cascaded response approaches a desired linear response with gain G. Figure 1 illustrates the system-level transmitter and feedback-synchronization context considered in this work. The PA output is observed through a directional coupler and a feedback receiver comprising attenuation, RF down-conversion, analog-to-digital converter (ADC) sampling, nominal time alignment, and gain normalization. The sampled feedback observation may contain a physical loop delay τ and additive observation noise w[n], where a positive τ denotes a delay of the feedback observation relative to the undelayed PA output. Nominal time alignment removes the dominant timing offset; however, analog propagation, sampling-clock uncertainty, and finite synchronization accuracy do not generally guarantee exact fractional-sample alignment. A residual fractional timing mismatch, denoted by δ, may therefore remain after nominal synchronization.

At the algorithm level, the DPD is optimized through a differentiable cascade consisting of the trainable predistorter and a fixed pretrained PA surrogate. For an input sequence x[n], the predistorter generates $z_{\theta }[n]=f_{DPD,\theta }(x[n])$, and the fixed PA surrogate subsequently produces ${\hat{y}}_{\theta }[n]=f_{PA}(z_{\theta }[n])$.

During DPD optimization, the parameters of the PA surrogate remain fixed, while gradients are propagated through the differentiable surrogate to update the DPD parameters. Defining the desired linear reference as r[n]=Gx[n], the DPD parameters are obtained by minimizing the mean-square cascade error:

(2) $$\theta *=\arg\;\underset{\theta }{\min}\frac{1}{N}\sum_{n=1}^{N}{\left|f_{PA}(f_{DPD,\theta }(x[n]))-Gx[n]\right|}^{2}$$

where θ denotes the trainable DPD parameters, θ* denotes their optimized values, N is the number of samples included in the loss evaluation, $f_{DPD,\theta }(\cdot )$ denotes the predistorter mapping, $f_{PA}(\cdot )$ denotes the fixed PA-surrogate mapping, and G is the desired linear gain.

Figure 1 therefore describes the practical observation and synchronization context, whereas Equation (2) defines the differentiable algorithm-level optimization used in this study. During optimization, the parameters of the PA surrogate remain fixed, while gradients are propagated through the surrogate to update only the DPD parameters. The sensitivity of this adaptation process to residual fractional timing mismatch after nominal synchronization is quantitatively investigated in Section 4.7. For temporal processing, the input sequence is subsequently windowed into multidimensional tensors and processed by the EA-CCF-AttGRU architecture described in Section 3.

## 3. EA-CCF-AttGRU Architecture

**Figure 2:**
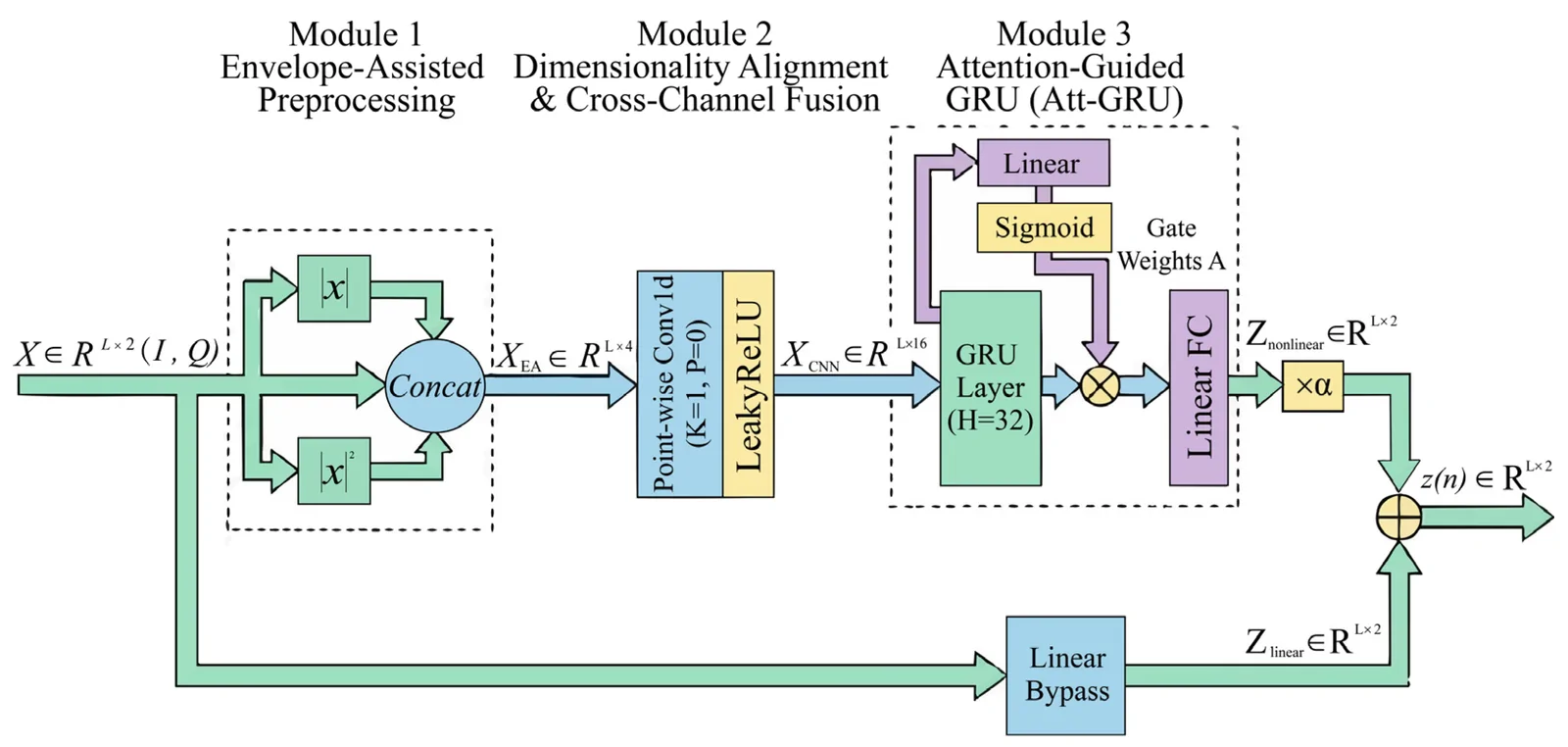
Detailed internal topology of the proposed single-box EA-CCF-AttGRU architecture. The arrows indicate the signal propagation direction between functional modules. Different colors represent different functional components, including envelope-assisted preprocessing, cross-channel feature fusion, attention-guided recurrent modeling, and linear bypass paths. Dashed boxes indicate the boundaries of individual processing modules. The circular nodes represent feature concatenation or residual addition operations. The original complex baseband input x is augmented by the Envelope-Assisted (EA) module, followed by point-wise cross-channel feature fusion and an Attention-Guided Gated Recurrent Unit (AttGRU) for temporal memory modeling. A global linear bypass is used for adaptive residual fusion.

To reduce the computational and structural burden of wideband neural DPD while maintaining a unified end-to-end mapping, a functionally decoupled single-box architecture, termed EA-CCF-AttGRU, is developed. Consistent with the functional role assignment introduced in Section 1, the architecture assigns envelope-assisted feature augmentation and strictly point-wise cross-channel fusion to instantaneous nonlinear feature construction, while temporal-memory evolution is handled by the subsequent attention-guided GRU. An independent global linear bypass provides a direct representation path for the dominant affine compensation component, allowing the recurrent nonlinear branch to focus on the remaining nonlinear and memory-dependent compensation. The point-wise CCF employs a kernel size of one together with a leaky rectified linear unit (LeakyReLU) activation, so that nonlinear cross-channel fusion is performed at each timestamp without explicit cross-timestamp mixing at this stage. The detailed signal flow and internal tensor operations are illustrated in Figure 2.

### 3.1. Envelope-Assisted Feature Augmentation Module

Directly learning the mapping from Cartesian in-phase/quadrature (I/Q) components to complex nonlinear distortion can increase the representational burden on a network in conventional baseband processing. Amplitude-dependent phenomena, such as amplitude-to-amplitude (AM-AM) and amplitude-to-phase (AM-PM) conversion, are closely related to the instantaneous signal envelope rather than to independent Cartesian components.

Envelope-derived features have been widely adopted in nonlinear PA modeling and DPD architectures, including Augmented Real-Valued Time-Delay Neural Network (ARVTDNN)-based approaches [5]. Therefore, the extraction of |x| and $|x{|}^{2}$ is not considered an independent contribution of this work. Instead, these established parameter-free features are employed as lightweight amplitude-related input augmentation before subsequent feature fusion. Within the proposed architecture, the role of the EA stage is to provide instantaneous envelope information to the point-wise CCF, rather than to introduce a new envelope-feature formulation. The resulting augmented representation is subsequently processed by the strictly point-wise CCF before temporal dependencies are introduced by the AttGRU. In this way, the EA stage provides instantaneous amplitude-related information for subsequent feature processing, while temporal dependencies are modeled by the recurrent stage within the same end-to-end predistorter.

Given an arbitrary complex baseband input sequence $X\in R^{L\times 2}$, the instantaneous envelope and squared-envelope features are defined as follows.

(3) $$|x[n]|=\sqrt{x_{I}^{2}[n]+x_{Q}^{2}[n]+\epsilon },\;\;|x[n]{|}^{2}=x_{I}^{2}[n]+x_{Q}^{2}[n],$$

Here, L denotes the temporal sequence length, $x_{I}[n]$ and $x_{Q}[n]$ denote the in-phase and quadrature components at time index n, respectively, and $\epsilon =10^{-8}$ is a small positive constant introduced for numerical stability during gradient backpropagation.

### 3.2. Point-Wise Cross-Channel Fusion and Dimensionality Alignment

At each time index n, the EA module forms the following instantaneous four-channel feature vector.

(4) $$f_{n}=[x_{I}[n],x_{Q}[n],|x[n]|,|x[n]{|}^{2}{]}^{T}$$

Here, $f_{n}\in R^{4}$ denotes the augmented feature vector at time index n, and the superscript T denotes matrix transpose.

To formalize the distinction between temporal and point-wise feature fusion, define the temporal-offset set as follows.

(5) $$T=\{\tau_{0},\tau_{1},\ldots ,\tau_{K-1}\}$$

Here, K>1 denotes the temporal-convolution kernel size, and $\tau_{k}$ denotes the temporal offset associated with the k-th kernel coefficient.

Using these offsets, the output of a temporal convolution at time index n can be represented as

(6) $$v_{n}=\psi (\sum_{k=0}^{K-1}W_{k}f_{n-\tau_{k}}+b).$$

Here, $v_{n}$ denotes the temporal-convolution output, $W_{k}$ is the learnable weight matrix associated with offset $\tau_{k}$, b is the corresponding bias vector, and ψ(⋅) denotes a point-wise nonlinear activation.

The same temporal dependency can be illustrated for a complex sequence written as

(7) $$x[n]=A[n]e^{j\phi [n]}$$

where A[n] and ϕ[n] denote the instantaneous amplitude and phase, respectively, and $j=\sqrt{-1}$.

A temporal linear combination has the form

(8) $$\tilde{x}[n]=\sum_{k=0}^{K-1}h_{k}x[n-\tau_{k}]=\sum_{k=0}^{K-1}h_{k}A[n-\tau_{k}]e^{j\phi [n-\tau_{k}]}.$$

Here, $h_{k}$ denotes the k-th temporal filter coefficient, and $\tilde{x}[n]$ denotes the resulting temporally mixed complex sample. This expression shows that the resulting value can depend on amplitudes and phases associated with multiple time indices.

This dependency alone does not imply that temporal convolution intrinsically degrades phase fidelity; its effect depends on the learned coefficients and the subsequent nonlinear mapping.

In contrast, the point-wise CCF used in the proposed architecture has a kernel size of one and operates only on the feature channels at the current time index:

(9) $$u_{n}=LeakyReLU(W_{CCF}f_{n}+b_{CCF})$$

Here, $u_{n}\in R^{16}$, $W_{CCF}\in R^{16\times 4}$, and $b_{CCF}\in R^{16}$ denote the CCF output, weight matrix, and bias vector, respectively.

Because $u_{n}$ contains no functional dependence on $f_{m}$ for m≠n, the corresponding cross-timestamp Jacobian satisfies the following relation wherever the activation is differentiable.

(10) $$\frac{\partial u_{n}}{\partial f_{m}}=0,m\neq n.$$

Here, m denotes an arbitrary timestamp different from n, and $0\in R^{16\times 4}0$ denotes the zero Jacobian matrix.

Therefore, the point-wise CCF performs instantaneous cross-channel fusion without explicit cross-timestamp mixing at this stage. This structural property does not imply that the complete EA-CCF-AttGRU network is temporally memoryless, nor does it mathematically guarantee preservation of complex phase. Temporal dependencies are subsequently introduced through the recurrent state transition,

(11) $$h_{n}=GRU(u_{n},h_{n-1})$$

where $h_{n}$ and $h_{n-1}$ denote the current and previous recurrent hidden states, respectively.

Accordingly, instantaneous cross-channel feature fusion and recurrent temporal modeling are assigned to different functional components within the same end-to-end architecture.

### 3.3. Channel-Wise Attention-Guided GRU

To model the temporal-memory evolution of the wideband PA within the proposed functional organization, the fused feature sequence is processed by a GRU. The recurrent state provides the temporal dependency that is intentionally excluded from the preceding point-wise CCF stage. Because the relative importance of recurrent hidden-state features can vary with the instantaneous signal condition and operating state, a lightweight channel-wise attention gate (CAG) is applied to the GRU outputs to provide data-dependent feature reweighting. In the proposed architecture, the attention gate is therefore used as an adaptive reweighting mechanism for the recurrent representation rather than as an independent temporal-memory modeling path.

Given the fused feature $X_{CNN}$ as input, let $H=GRU(X_{CNN})\in R^{L\times C_{h}}$ denote the sequential hidden states produced by the GRU layer. The attention mechanism computes a data-driven weight matrix $A\in R^{L\times C_{h}}$ to adaptively scale the features:

(12) $$A=\sigma (HW_{gate}^{T}+1_{L}b_{gate}^{T})$$

(13) $$H_{attended}=A⊙H$$

where $W_{gate}\in R^{C_{h}\times C_{h}}$ and $b_{gate}\in R^{C_{h}}$ are the learnable weight matrix and bias vector of the attention linear layer, $1_{L}\in R^{L}$ is an all-ones vector used to broadcast the bias over the temporal dimension, σ(⋅) denotes the sigmoid activation function, and ⊙ denotes the element-wise Hadamard product.

A subsequent fully connected layer projects $H_{attended}$ onto the two-channel nonlinear compensation vector $Z_{nonlinear}\in R^{L\times 2}$. With respect to the sequence length L, the channel-wise gating operation scales linearly in L for a fixed hidden dimension $C_{h}$, with a computational complexity of $O(LC_{h}^{2})$. In contrast, standard sequence-wise self-attention exhibits a computational complexity of $O(L^{2}C_{h})$ because of the pairwise attention matrix.

### 3.4. Global Linear Bypass and Adaptive Residual Fusion

Because the PA fundamentally operates as an amplifier, the predistorted signal remains strongly linearly correlated with the input baseband sequence. Requiring the nonlinear branch to represent this linear transformation in addition to nonlinear and memory-dependent effects can increase its representational burden.

An independent affine transformation layer captures the dominant linear gain and phase transformation directly from the original I/Q input tensor $X\in R^{L\times 2}$:

(14) $$Z_{linear}=XW_{bypass}^{T}+1_{L}b_{bypass}^{T}$$

where $Z_{linear}\in R^{L\times 2}$ denotes the output tensor of the linear bypass, $W_{bypass}\in R^{2\times 2}$ and $b_{bypass}\in R^{2}$ are the learnable weight matrix and bias vector of the affine transformation, respectively, and $1_{L}\in R^{L}$ is an all-ones vector used to broadcast the bias across the L timestamps. The superscript T denotes matrix transpose.

The final predistorted output tensor $Z\in R^{L\times 2}$ is obtained by combining the linear-bypass output with the adaptively scaled nonlinear compensation:

(15) $$Z=Z_{linear}+\alpha Z_{nonlinear}.$$

where $Z_{nonlinear}\in R^{L\times 2}$ denotes the two-channel nonlinear compensation tensor generated by the main neural branch, and α∈R is a learnable residual scaling coefficient initialized to 1.0. The additive linear bypass provides a direct representation path for the dominant linear component, allowing the recurrent nonlinear branch to focus on the remaining nonlinear and memory-dependent compensation. The coefficient α is jointly optimized with the other DPD parameters to adapt the relative contribution of the nonlinear compensation term.

### 3.5. Hardware-Aware Computational Complexity and Training Strategy

In highly oversampled wideband applications, multiplication and addition counts alone do not fully characterize the implementation burden of a neural DPD model. We therefore extend the per-sample analysis to dense arithmetic, element-wise arithmetic, nonlinear and special-function evaluations, and memory/state requirements. Nonlinear functions and special operations are reported separately rather than being assigned arbitrary equivalent arithmetic costs. These quantities provide a hardware-aware analytical description of the forward inference path, but they do not represent measured hardware latency, throughput, resource utilization, or timing closure. Actual implementation performance additionally depends on arithmetic precision, resource mapping, clock frequency, pipelining strategy, and the initiation interval (II) of the recurrent datapath.

Table 1 summarizes the hardware-aware analytical profiles of the three models used in the iso-parameter comparison. The proposed EA-CCF-AttGRU uses H=32 with 6009 parameters, while the long short-term memory (LSTM) uses H=37 with 6144 parameters and the temporal convolutional network (TCN) uses H=207 with 6003 parameters. The operator counts are derived from the corresponding model dimensions, while the TCN temporal-buffer requirement is reported separately as an analytical estimate for a straightforward streaming realization.

In addition to the operations listed in Table 1, the point-wise CCF stage contains 16 LeakyReLU evaluations per sample, while the TCN contains 1035 Hardswish evaluations per sample. Table 1 shows that the three iso-parameter models have similar parameter storage and dense arithmetic workloads, but their computational profiles differ in nonlinear operations, temporal state, and routing width. Compared with the LSTM, the proposed EA-CCF-AttGRU reduces the persistent recurrent state from 74 to 32 values and the logical recurrent-state read/write requirement from 148 to 64 values per sample. The proposed model also requires 96 Sigmoid and 32 Tanh evaluations per sample, compared with 111 Sigmoid and 74 Tanh evaluations for the LSTM.

The TCN eliminates recurrent feedback but uses a substantially wider principal feature path of 207 channels. It also contains 1035 Hardswish evaluations, one square-root operation, and two divisions per sample. For a straightforward streaming realization of its four dilated depthwise convolutional layers, the temporal delay-line requirement is analytically estimated as 12,420 values. Therefore, the three architectures exhibit different trade-offs among recurrent state, temporal buffering, feature width, and nonlinear operations. These quantities provide a hardware-aware analytical comparison and are not interpreted as synthesized digital signal processing (DSP), look-up table (LUT), flip-flop (FF), BRAM, latency, or II results.

To complement the analytical operator and state counts with a practical software-level breakdown, the forward path of EA-CCF-AttGRU was profiled using PyTorch 2.5.1 on an NVIDIA GeForce RTX 3080 Ti GPU platform. As summarized in Table 1c, for a sequence length of 16,384 samples, the median execution times of the EA feature extraction, point-wise CCF with LeakyReLU, GRU, attention gate, and output/bypass/fusion stages were 0.337, 0.234, 6.758, 0.251, and 0.218 ms, respectively, while the complete forward pass required 7.336 ms. The GRU therefore dominates the measured software execution time for long sequences. For the shorter 200-sample sequence, the complete forward pass required 1.454 ms; at this sequence length, individual module timings are more strongly influenced by software and kernel-launch overhead. Because the module timings and the full-forward timing were measured separately, the individual values are not expected to sum exactly to the full-forward value. These measurements characterize software execution on the tested GPU platform and are not interpreted as FPGA/ASIC pipeline latency, initiation interval, maximum clock frequency, or timing-closure results.

During DPD optimization, the pretrained PA surrogate is kept fixed, and the mean-squared error (MSE) defined in Equation (2) between the cascade output and the desired linear reference is minimized using AdamW. Gradients are propagated through the differentiable PA surrogate, but only the DPD parameters are updated. Because backpropagation through time (BPTT) is considerably more expensive than forward inference, practical online adaptation may employ block-wise or intermittently scheduled updates. Section 4.8 therefore quantifies the algorithm-level responsiveness–update-workload trade-off for block sizes from 4096 to 65,536 samples under controlled dynamic power transitions.

## 4. Experimental Results and Analysis

### 4.1. Experimental Setup and Dual-Track Benchmarking

Evaluating neural digital predistortion architectures under wideband memory effects requires a standardized and reproducible benchmarking environment. The proposed architecture was therefore evaluated using the open-source OpenDPD learning and benchmarking framework [26]. Specifically, the experiments employed the DPA_160MHz dataset, which contains nonlinear behavioral data from a 40 nm complementary metal–oxide–semiconductor (CMOS) digital power amplifier (DPA) operating at a 2.4 GHz carrier frequency.

The excitation consisted of four aggregated 40 MHz carriers, resulting in a total signal bandwidth of 160 MHz. The complex baseband waveform was sampled at 640 MS/s ($f_{s}=640$ MHz), corresponding to an inter-sample interval of $T_{s}=1/f_{s}=1.5625$ ns. An OFDM scheme without a cyclic prefix and 1024-QAM modulation was employed for signal generation, with 1024 active subcarriers per carrier and an inverse fast Fourier transform (IFFT) frame size of 16,384. The resulting baseband sequence had a PAPR of 10.38 dB.

As shown in Table 2, the combination of a 160 MHz wideband envelope and a 10.38 dB PAPR repeatedly drove the power amplifier into saturation. This excitation resulted in a severe dynamic electrical memory effect and significant out-of-band spectral regrowth; and thus provided a demanding dynamic environment for testing linearization limits.

For neural-network training, the continuous baseband stream was dynamically windowed into temporal tensors with a sequence length of L=200 discrete samples. The sequence length is defined in sample units and does not represent an independent sampling rate or a hardware computation period. This temporal depth was selected to provide sufficient context for modeling the dominant electrical memory effects of the DPA while maintaining stable BPTT optimization. Following the OpenDPD protocol, the dataset was divided into 60% training, 20% validation, and 20% independent testing subsets.

End-to-end DPD optimization requires a differentiable forward representation of the PA so that gradients can be propagated from the cascade output to the predistorter. Before evaluating the DPD models, a high-fidelity digital twin of the wideband DPA was therefore pretrained using a GRU backbone. As described in Section 2.1, this forward behavioral model achieved an NMSE of −38.45 dB. The same pretrained PA surrogate was subsequently kept fixed for all DPD training and robustness experiments, ensuring that the evaluated models were compared under an identical forward nonlinear environment.

To isolate the structural approximation capability of the evaluated neural networks from external hardware impairments, a noise-free floating-point digital-twin environment is employed. Accordingly, the reported NMSE and EVM values characterize the noise-free algorithmic approximation performance under the adopted digital-twin environment. In a physical RF testbed, these metrics would additionally be affected by thermal noise, ADC quantization errors, and local-oscillator phase noise.

To reduce the influence of model capacity on architectural comparison, this study employed a two-track benchmarking methodology comprising default-capacity and approximately iso-parameter configurations. RVTDCNN [31], LSTM [32], and TCN-DPD [24] were first evaluated using lightweight default configurations and were then scaled to approximately 6000 trainable DPD parameters. Strictly counting only the trainable DPD network and excluding the 1911 parameters of the fixed PA digital twin, their default configurations contain 617, 1172, and 435 parameters, respectively. To extend the comparison to more recent recurrent DPD designs, APNRRU [28], DeltaGRU [30], and TRes-DeltaGRU [30] were evaluated using the same two-track protocol. Their default configurations use a hidden size of 15 and contain 1393, 1067, and 999 trainable DPD parameters, respectively. Their capacity-scaled configurations use hidden sizes of 81, 41, and 42, resulting in 6013, 6111, and 6156 trainable DPD parameters, respectively. For DeltaGRU and TRes-DeltaGRU, the delta thresholds were set to zero in both configurations so that the comparison characterizes their dense recurrent baselines without sparsity-induced operation skipping. The generalized memory polynomial (GMP) [3] was configured with 495 parameters.

Table 3 outlines both the default-capacity and capacity-scaled configurations. The selected EA-CCF-AttGRU configuration contains 6009 trainable parameters. Matching this approximate parameter range requires the LSTM to expand to 37 hidden units (6144 parameters), the RVTDCNN to 156 hidden channels (6116 parameters), and the TCN-DPD to 207 hidden channels (6003 parameters). This approximately iso-parameter comparison substantially controls differences in trainable model size and therefore provides a more direct assessment of whether the observed performance differences are not solely attributable to parameter count under comparable parameter budgets.

PyTorch 2.5.1 was used to build and train the models. The empirical optimization reduced the MSE loss function for mini-batches of 256 samples. AdamW optimizer and a step-decay learning rate scheduler (StepLR) were used for training execution. The initial learning rate was 0.0005, and every 30 epochs for the course of 150 epochs, it was reduced by a factor of 0.5. The residual scaling coefficient α was initialized to 1.0 and jointly optimized with the remaining DPD parameters during training.

The original primary comparison among RVTDCNN, LSTM, TCN, and EA-CCF-AttGRU was repeated using five random seeds. The observed NMSE and ACLR standard deviations were below 0.1 dB, and the corresponding mean values are reported. The newly added APNRRU, DeltaGRU, and TRes-DeltaGRU architectures were each trained once in both the default-capacity and iso-parameter configurations under the same DPA_160MHz data split, fixed PA surrogate, temporal frame length, optimizer schedule, and validation-based checkpoint-selection procedure. These six newly added entries are therefore reported as single-run results and are not included in the five-seed statistics of the original primary comparison. In Section 4.7, five matched adaptation runs were performed for each residual timing-offset condition, and the corresponding robustness results are reported as the mean ± one standard deviation.

**Figure 3:**
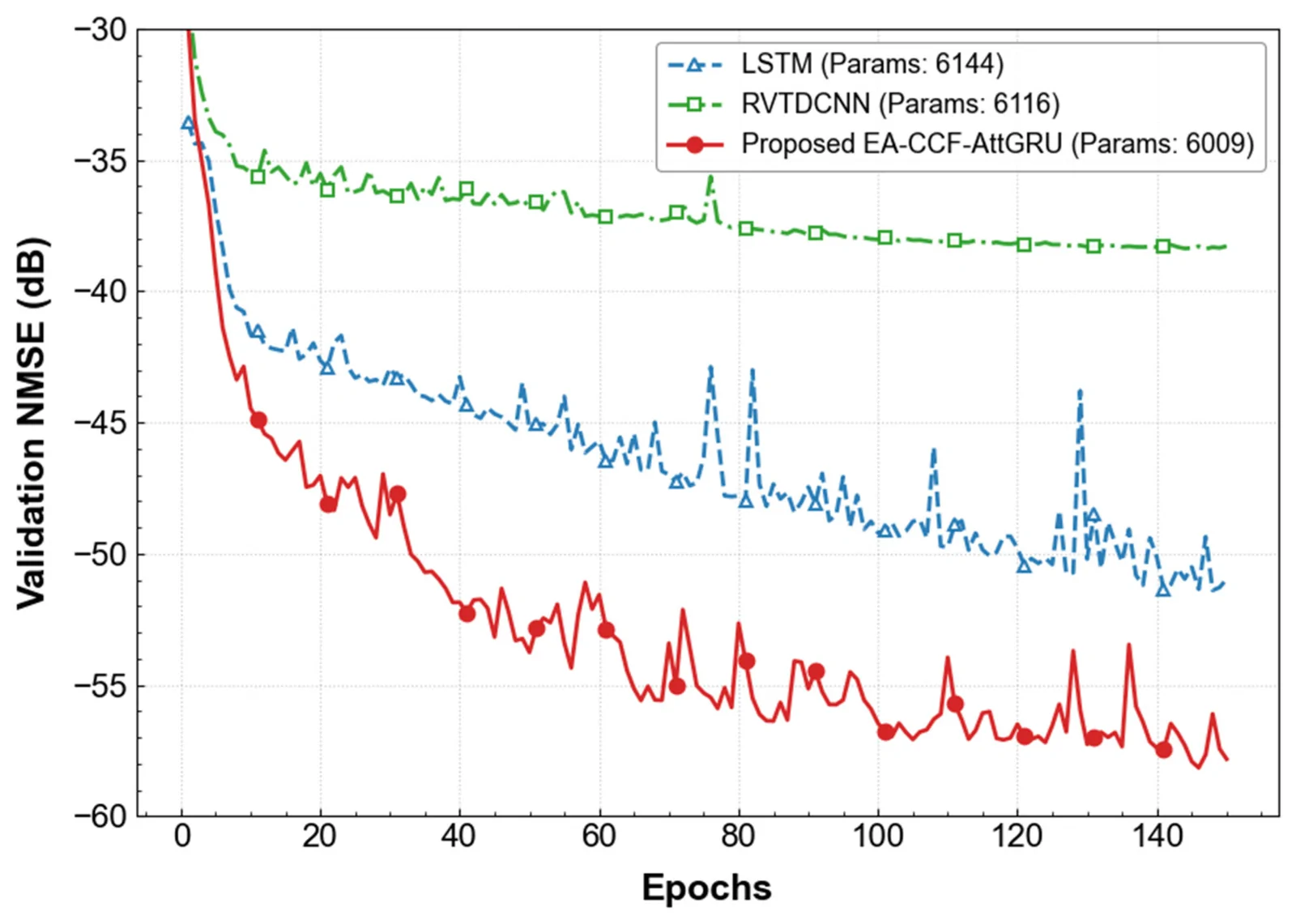
Convergence Trajectories of the Validation NMSE over 150 Training Epochs under the iso-parameter Setup (160 MHz, 1024-QAM). The base learning rate of 0.0005 decayed by a factor of 0.5 every 30 epochs. The proposed EA-CCF-AttGRU (red solid line, 6009 parameters) showed fast convergence at decay steps and outperformed the baseline RVTDCNN (green dash-dotted line, 6116 parameters) and LSTM (blue dashed line, 6144 parameters) networks.

The validation trajectories of the approximately iso-parameter configurations are shown in Figure 3. The proposed EA-CCF-AttGRU reaches a validation NMSE near −57.84 dB and exhibits stable convergence over the adopted 150-epoch optimization schedule. Under comparable parameter budgets, its validation trajectory reaches a lower residual-error region than those of the scaled LSTM and RVTDCNN baselines shown in Figure 3. These results characterize the optimization behavior of the evaluated configurations under the common training protocol and complement the independent test-set comparisons reported in Section 4.2.

### 4.2. Comprehensive Linearization Performance Analysis

**Figure 4:**
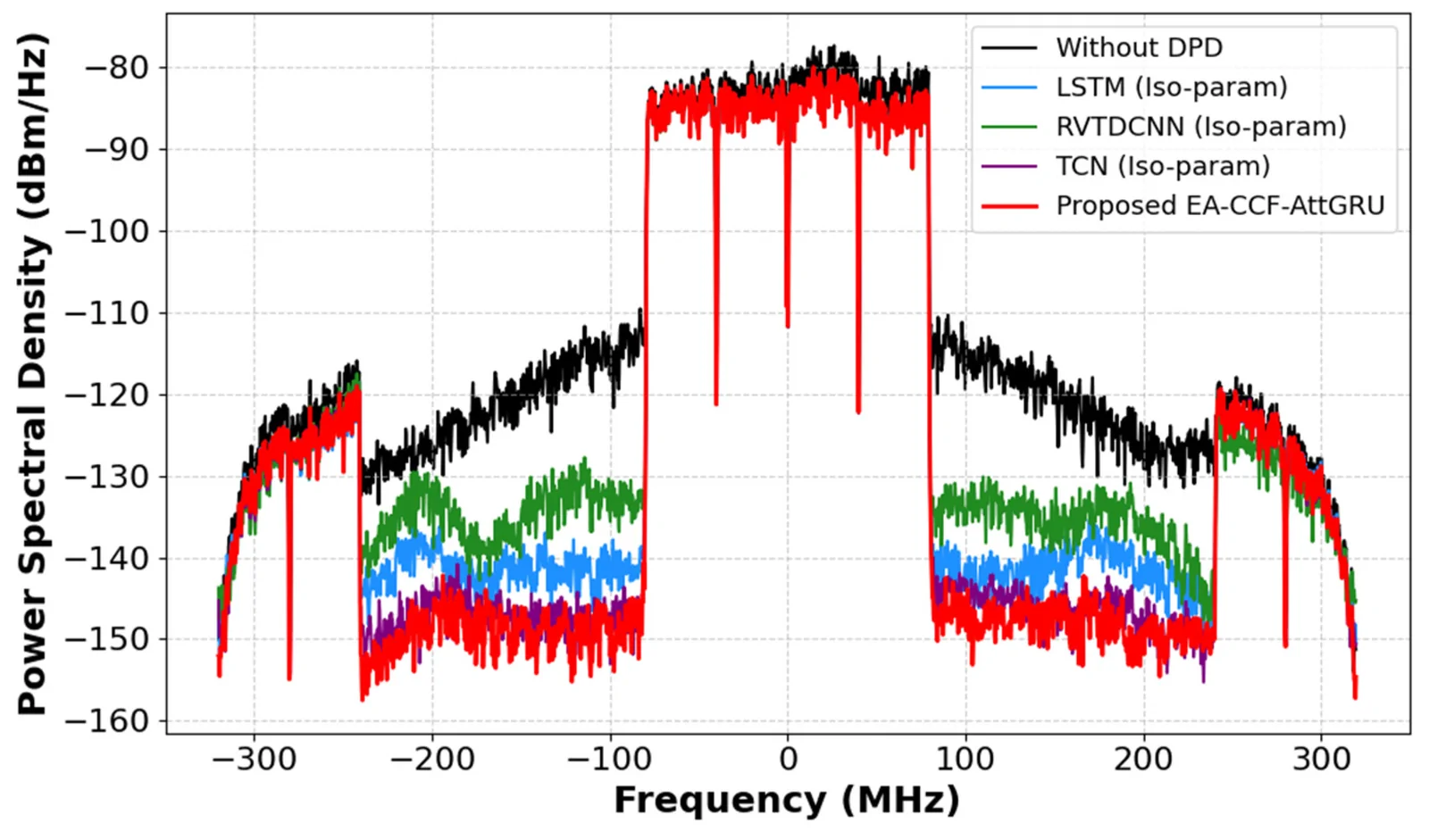
Comparison of PSD under 160 MHz wideband excitation (1024-QAM, PAPR = 10.38 dB). At an iso-parameter constraint of approximately 6000 parameters, the proposed EA-CCF-AttGRU (red line) suppresses out-of-band spectral regrowth to an ACLR of −65.91 dBc and outperforms the uncompensated PA (Without DPD, black line), the scaled baseline models (LSTM, blue line; RVTDCNN, green line), and the iso-parameter TCN (purple line, −62.93 dBc). The deep in-band spectral notches are the direct-current (DC) and null subcarriers that naturally occur in the 4-carrier OFDM frame structure.

Validation of the linearization effect requires examination of both frequency-domain spectral regrowth and time-domain nonlinear distortion. Figure 4 is the Power Spectral Density (PSD) of the PA output. Using a 160 MHz wideband test signal, the uncompensated PA (without DPD) has relatively large out-of-band emissions. Under the strict two-track evaluation system, the extended traditional baseline model at the iso-parameter boundary has achieved bounded spectral correction. The iso-parameter RVTDCNN, LSTM and TCN saturate at ACLR values of −50.87 dBc, −58.85 dBc and −62.93 dBc respectively. The proposed EA-CCF-AttGRU substantially suppresses out-of-band spectral regrowth and achieves an ACLR of −65.91 dBc. Therefore, there is a performance gap; this confirms that the coordinated design of envelope-assisted feature augmentation and point-wise dimensionality alignment effectively improves high-order nonlinear representation without relying solely on parameter accumulation.

Validation of the linearization performance requires examination of both frequency-domain spectral regrowth and time-domain nonlinear distortion. Figure 4 compares the power spectral density (PSD) of the uncompensated PA output and the evaluated predistorted outputs under the 160 MHz wideband excitation. The uncompensated PA exhibits pronounced out-of-band spectral regrowth. Under the approximately iso-parameter evaluation, the RVTDCNN, LSTM, and TCN-DPD baselines achieve ACLR values of −50.87, −58.85, and −62.93 dBc, respectively, whereas the proposed EA-CCF-AttGRU achieves −65.91 dBc. The persistence of this performance advantage under comparable parameter budgets provides controlled evidence that the observed improvement is not explained by trainable parameter count alone. The component-level origin of this advantage is examined separately through the ablation and architectural-variant analysis in Section 4.4.

**Figure 5:**
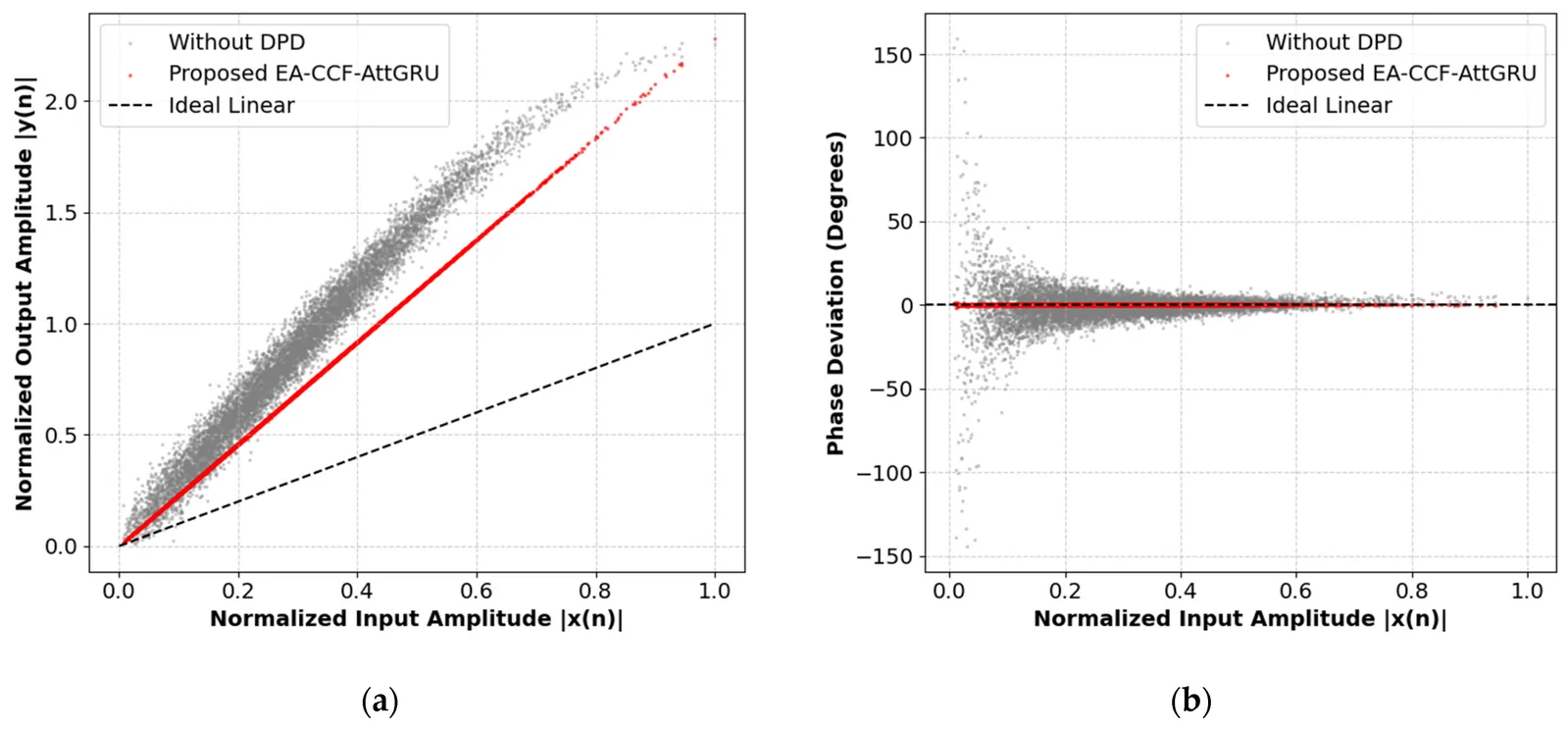
(**a**) Normalized output amplitude (AM-AM) and (**b**) phase shift (AM-PM) as functions of normalized input amplitude under 160 MHz wideband excitation (1024-QAM, PAPR = 10.38 dB) in the time domain. The dispersed response of the uncompensated PA (black dots) is consistent with pronounced dynamic memory effects and static amplitude compression. In contrast, the proposed EA-CCF-AttGRU (red dots) concentrates the responses around the desired linear trajectory while substantially reducing the residual phase dispersion.

Figure 5 presents the AM-AM and AM-PM scatter characteristics. The uncompensated PA exhibits a widely dispersed response cloud, reflecting pronounced dynamic memory effects and static amplitude compression. With EA-CCF-AttGRU predistortion, the responses become concentrated around the desired linear AM-AM trajectory, while the AM-PM dispersion is substantially reduced. These time-domain observations are consistent with the frequency-domain linearization results in Figure 4 and provide empirical evidence of improved compensation under the 160 MHz wideband excitation.

Table 4 summarizes the linearization performance of the evaluated architectures under both default-capacity and approximately iso-parameter settings. The recent recurrent baselines exhibit different capacity-scaling behaviors under the common evaluation protocol. APNRRU achieves an NMSE/ACLR of −45.82 dB/−54.83 dBc in its 1393-parameter default configuration and −45.78 dB/−53.75 dBc after scaling to 6013 parameters, indicating no improvement from the added capacity under the present training configuration. DeltaGRU improves from −48.72 dB/−57.77 dBc with 1067 parameters to −56.14 dB/−63.94 dBc with 6111 parameters, while TRes-DeltaGRU improves from −51.81 dB/−59.33 dBc with 999 parameters to −56.97 dB/−64.51 dBc with 6156 parameters. Under the same DPA_160MHz evaluation environment, the proposed EA-CCF-AttGRU achieves −57.84 dB NMSE, 0.07% EVM, and −65.91 dBc ACLR with 6009 trainable parameters. Thus, within the evaluated approximately 6000-parameter range, the proposed architecture achieves the lowest NMSE and most negative ACLR among the compared configurations while maintaining a comparable trainable parameter budget.

### 4.3. Capacity Scaling and Observed Complexity–Performance Knee Point

Varying the hidden dimension provides an empirical characterization of model-capacity scaling under the adopted benchmark. Increasing the hidden dimension enlarges the available representational capacity, although the resulting linearization improvement depends on how effectively each architecture uses the additional parameters. The iso-parameter results show that the TCN-DPD baseline benefits substantially from capacity expansion: increasing its parameter count from 435 to 6003 improves ACLR from −54.95 to −62.93 dBc and NMSE from −48.55 to −56.40 dB. This result indicates that the scaled TCN-DPD uses the expanded parameter budget effectively under the present evaluation.

The proposed EA-CCF-AttGRU nevertheless achieves −65.91 dBc ACLR and −57.84 dB NMSE with 6009 parameters. Increasing its hidden dimension further raises the parameter count to 20,121 and improves ACLR to −66.89 dBc. The additional 14,112 parameters therefore provide only a further 0.98 dB ACLR improvement under the evaluated configuration, indicating diminishing marginal return beyond the selected 6009-parameter operating point.

The observed scaling behavior is also consistent with the functional role of the global linear bypass described in Section 3.4. As defined in Equation (15), the bypass provides a direct affine representation path for the dominant linear compensation component, while the recurrent nonlinear branch models the remaining nonlinear and memory-dependent compensation. This organization provides a structured separation between the dominant linear path and the nonlinear recurrent path, but it does not impose a mathematical constraint on how the nonlinear branch scales with hidden dimension. Under the evaluated configurations, increasing the model to 20,121 parameters improves ACLR to −66.89 dBc, while the marginal improvement remains small relative to the associated increase in model size.

For the DPA_160MHz dataset, the complex baseband stream is sampled at 640 MS/s, corresponding to an inter-sample interval of 1.5625 ns. This interval describes the temporal spacing between consecutive input samples and is not interpreted here as the measured end-to-end inference latency of the neural network.

**Figure 6:**
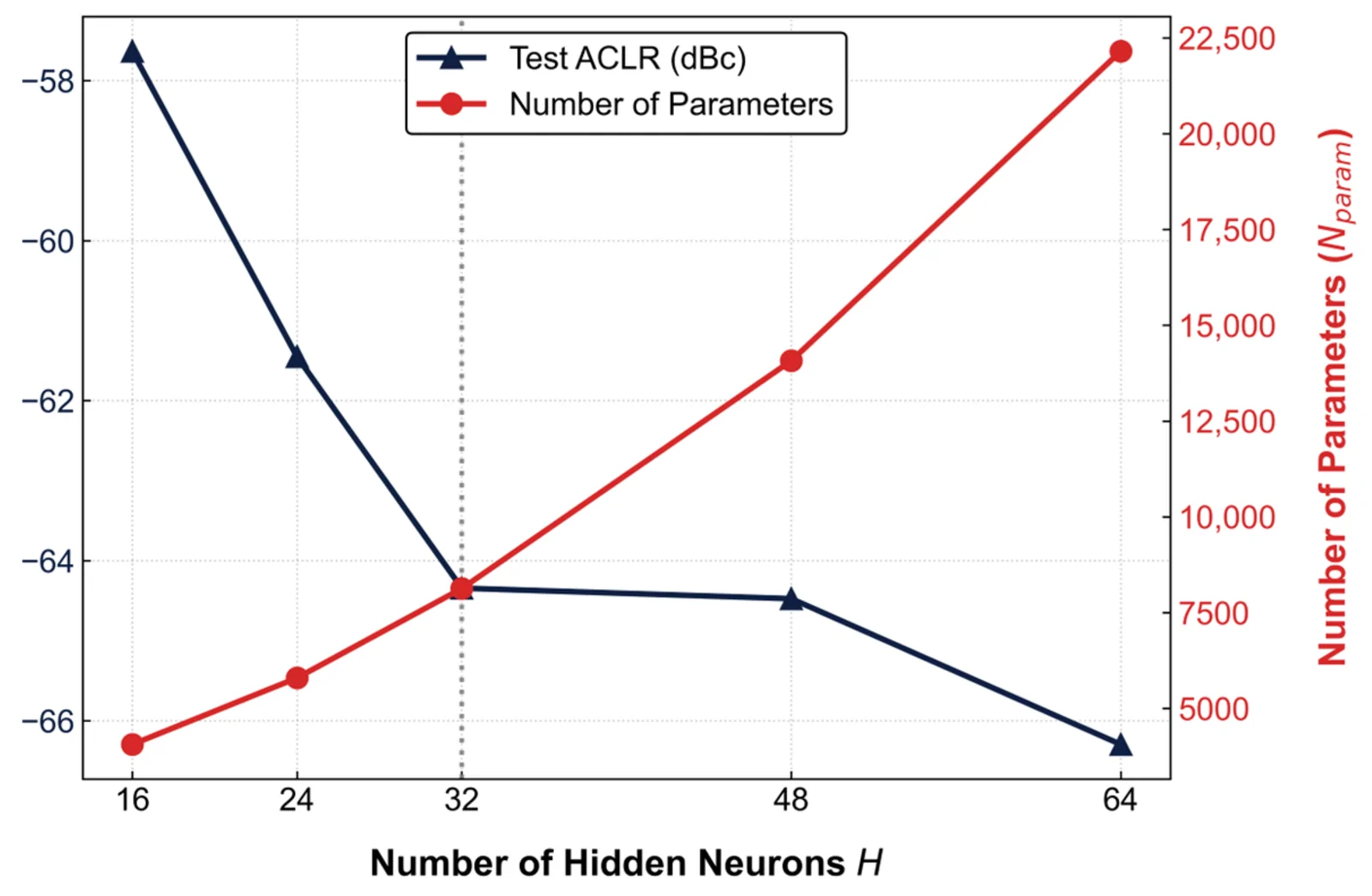
Dual-axis analysis of the scaling behavior of the proposed EA-CCF-AttGRU across different hidden dimensions under 160 MHz excitation (1024-QAM, PAPR = 10.38 dB). The left y-axis denotes the test ACLR, and the right y-axis denotes the pure DPD parameter count.The vertical dashed line marks the selected H=32 configuration, which contains 6009 trainable DPD parameters and represents the observed algorithmic complexity–performance knee point. Further expansion to H=64 increases the parameter count to 20,121 while providing only an additional 0.98 dB ACLR improvement.

Figure 6 is therefore interpreted from an algorithmic complexity–performance perspective. At H=32, the proposed EA-CCF-AttGRU achieves an ACLR of −65.91 dBc using 6009 trainable DPD parameters. Increasing the hidden dimension to H=64 increases the parameter count to 20,121 while improving ACLR to −66.89 dBc. Thus, increasing the model size by more than threefold beyond H=32 provides an additional ACLR improvement of only 0.98 dB. On this basis, H=32 is identified as the observed algorithmic complexity–performance knee point rather than a hardware timing boundary. The hardware-aware results in Section 3.5 characterize the operator composition, state requirement, and routing width of the selected H=32 configuration; however, these analytical quantities do not determine physical throughput.

For a future pipelined hardware realization, processing the 640-MS/s input stream would require a sustained throughput of at least 640 MS/s. For a single-lane pipeline, the nominal sample throughput can be expressed as $R_{throughput}=f_{clk}/II$, where $f_{clk}$ denotes the hardware clock frequency. The sequential hidden-state dependency of the GRU may constrain the achievable II. Since FPGA/ASIC synthesis and timing analysis are outside the scope of the present study, no specific nanosecond-level inference latency or timing-closure claim is made.

### 4.4. Ablation and Architectural-Variant Study

To determine whether the functional roles assigned in Section 3 provide measurable benefit within the complete architecture, four leave-one-component-out ablations (Models A–D) were constructed by removing the EA augmentation, point-wise CCF, attention gate, or global linear bypass/residual-fusion path, respectively. In addition, Model E was included as an architectural variant by adding a parallel dilated temporal-convolution branch to the point-wise CCF path. All variants use the same base recurrent hidden dimension, while their parameter counts are allowed to change naturally according to the corresponding architectural modification. This design separates component-removal ablations from the alternative dual-track configuration and enables the resulting performance changes to be interpreted together with analytical computational cost. Table 5c therefore reports the relative changes in dense MAC count and FP32 parameter memory, together with the ACLR degradation of each variant relative to the complete EA-CCF-AttGRU configuration.

The normalized results in Table 5c make the performance–cost contribution of each architectural component more explicit. Removing the EA module reduces the dense-MAC workload and FP32 parameter memory by only 0.56% and 0.53%, respectively, while degrading ACLR by 2.64 dB. Removing the point-wise CCF provides the largest cost reduction, decreasing dense MACs by 21.10% and parameter memory by 20.50%, at an ACLR penalty of 0.96 dB. Removing the attention gate reduces dense MACs and parameter memory by 17.77% and 17.57%, respectively, but causes a 2.75 dB ACLR degradation. In contrast, removing the bypass/residual-fusion path saves only 0.07% of the dense MACs and 0.12% of the parameter memory while degrading ACLR by 2.77 dB, indicating a particularly favorable marginal performance–cost contribution of this path. The dual-track variant increases dense MACs and parameter memory by 3.33% and 3.46%, respectively, while its ACLR is 1.60 dB worse than that of the full model.

Model A (without EA feature augmentation): Removing the explicit |x| and $|x{|}^{2}$ envelope features eliminates direct amplitude-related information from the augmented input. Although this modification provides only a marginal reduction in arithmetic and parameter-memory cost, the NMSE and ACLR both deteriorate, indicating that the envelope-derived augmentation provides a useful performance contribution under the evaluated configuration.

Model B (without CCF): The point-wise CCF and its associated activation are removed, and the four-dimensional EA feature vector is fed directly to the GRU. This variant provides the largest reduction in dense MAC count and parameter memory among the component-removal ablations, while incurring only a 0.96 dB ACLR penalty. These results indicate that the point-wise CCF provides a useful instantaneous cross-channel transformation, although its contribution is accompanied by a measurable arithmetic cost.

Model C (without Attention Gate): Removing the channel-wise attention gate reduces both the dense-MAC workload and the number of Sigmoid evaluations, but the NMSE and ACLR deteriorate to −55.04 dB and −63.16 dBc, respectively. Together with the relative-cost results in Table 5c, this indicates that the attention gate provides a measurable linearization benefit at a moderate additional computational cost.

Model D (without Global Linear Bypass and Adaptive Residual Fusion): Removing the affine linear bypass and the learnable residual scaling coefficient produces only negligible reductions in dense MAC count and parameter memory, whereas the ACLR degrades by 2.77 dB. The bypass/residual-fusion path therefore provides a substantial linearization contribution at a very small marginal analytical cost. Because the bypass and residual scaling coefficient are removed jointly in Model D, their individual contributions cannot be separated by this ablation.

**Figure 7:**
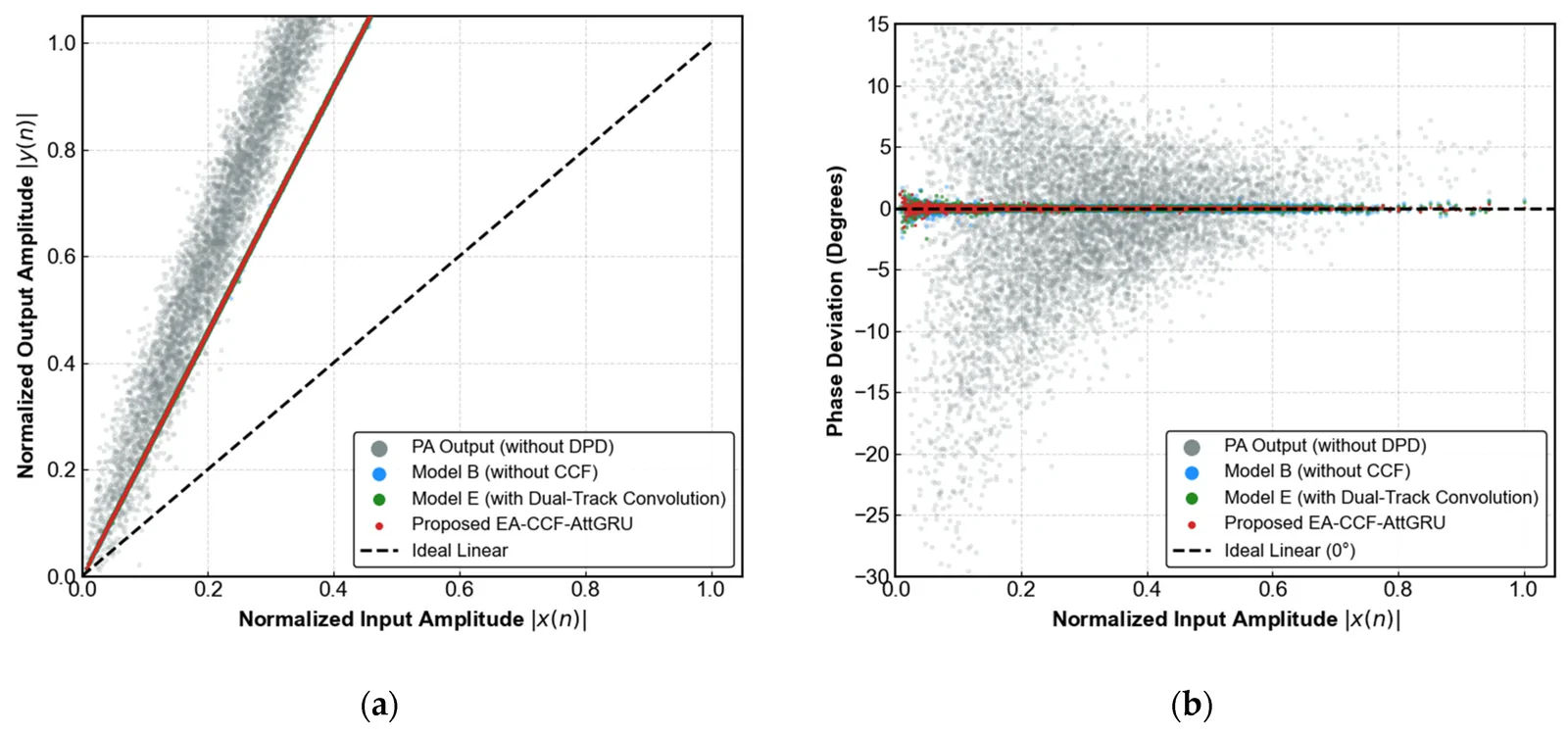
Visual comparison of the principal ablation variants under the 160 MHz wideband excitation (1024-QAM). (**a**) Normalized output amplitude (AM-AM) scatter plot. The uncompensated PA exhibits severe amplitude dispersion, whereas Model B, Model E, and the proposed EA-CCF-AttGRU compress the responses toward the ideal linear trajectory. (**b**) Phase shift (AM-PM) scatter plot. In the high-powesr region defined by |x(n)|>0.6, the measured phase-error standard deviations are approximately $0.24^{\circ }$, $0.14^{\circ }$, and $0.06^{\circ }$ for Model B, Model E, and the proposed configuration, respectively. These values are reported as empirical characteristics of the evaluated architectures and are not interpreted as evidence of a universal causal relationship between temporal convolution and phase distortion.

Model E (with Dual-Track Convolution): Adding a parallel dilated temporal-convolution branch increases the dense-MAC workload and FP32 parameter memory by 3.33% and 3.46%, respectively, while the ACLR deteriorates by 1.60 dB relative to the full model. Under the present experimental configuration, the additional temporal branch therefore increases the analytical implementation cost without improving the overall linearization performance. The residual phase dispersion shown in Figure 7 is reported only as an empirical characteristic of this specific configuration and is not interpreted as evidence that temporal convolution intrinsically causes phase distortion.

To visualize the empirical differences among the principal ablation variants, Figure 7 compares their time-domain AM-AM and AM-PM characteristics. As shown in Figure 7a, the uncompensated PA output exhibits severe dynamic-memory dispersion and static amplitude compression. Model B, Model E, and the proposed EA-CCF-AttGRU all substantially compress the amplitude responses toward the ideal linear trajectory, although the amplitude-domain result alone does not characterize the remaining phase behavior.

Figure 7b further compares the residual phase dispersion. In the high-power region defined by |x(n)|>0.6, the measured phase-error standard deviations are approximately $0.24^{\circ }$ for Model B, $0.14^{\circ }$ for Model E, and $0.06^{\circ }$ for the proposed configuration. These measurements show that the proposed configuration exhibits the smallest residual phase dispersion among the evaluated variants within this operating region. Together with the structural analysis in Section 3.2, these results provide empirical evidence for the selected architecture; however, they do not establish a universal causal relationship between temporal convolution and phase distortion.

Having established the contribution and computational cost of the principal architectural components, Section 4.5, Section 4.6, Section 4.7, Section 4.8 and Section 4.9 further examine the behavior of the proposed DPD under changes in operating power, feedback noise, synchronization mismatch, block-wise adaptation, and numerical precision.

### 4.5. Static Output-Scaling Behavior Under Fixed Power Back-Off

Practical transmitters operate over different input-power levels, requiring the predistorter to maintain appropriate output scaling over a range of excitation amplitudes. To characterize this behavior without parameter adaptation, the trained EA-CCF-AttGRU was evaluated separately at fixed input-power back-off levels of 0, −3, and −6 dB, while all model parameters were held unchanged.

**Figure 8:**
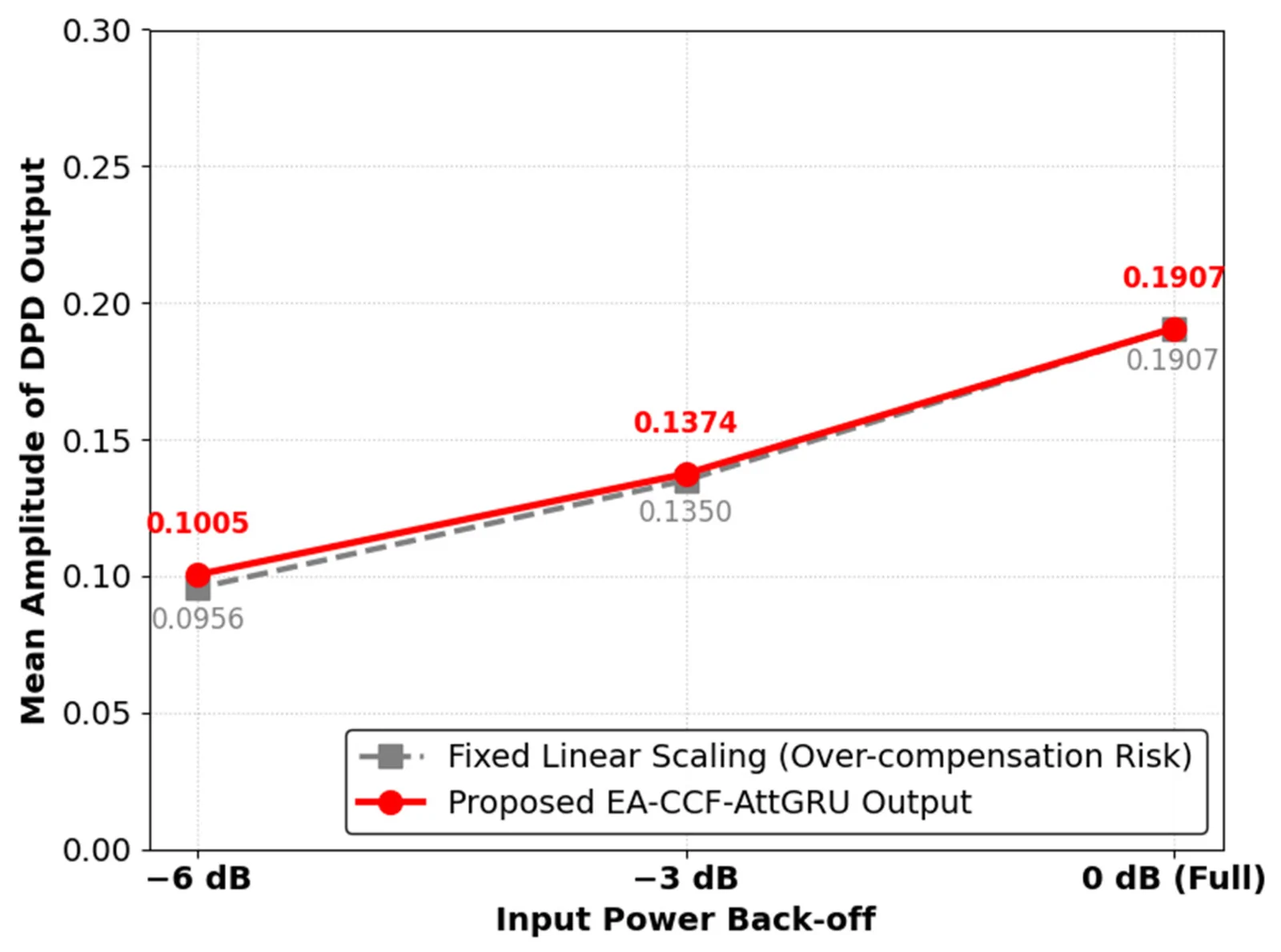
Mean amplitude of the DPD output as a function of input power back-off under 160 MHz wideband excitation (1024-QAM, PAPR = 10.38 dB). The proposed EA-CCF-AttGRU (red solid line with circular markers) produces corresponding output amplitudes at −3 dB and −6 dB back-off and remains close to the corresponding fixed linear-scaling reference (gray dashed line with square markers), indicating consistent output-scaling behavior across the evaluated back-off conditions.

Figure 8 illustrates the output-scaling behavior of the proposed architecture at 0, −3, and −6 dB input power back-off. A fixed linear scaling trajectory is used as the reference. With all model parameters held unchanged, the EA-CCF-AttGRU produces input-dependent output amplitudes as the excitation power changes. At 0 dB, the mean DPD output amplitude is 0.1907; at −3 and −6 dB, the corresponding values are 0.1374 and 0.1005, respectively. These values are close to the linear-scaling references of 0.1350 and 0.0956 for −3 and −6 dB, respectively, indicating consistent fixed-parameter output scaling across the evaluated power-back-off conditions.

### 4.6. Robustness to Controlled Feedback-Noise Perturbations

Section 4.5 characterizes the fixed-parameter output-scaling behavior of the proposed model under different input power-back-off conditions. In practical DPD systems, the feedback observation used for adaptation and evaluation can additionally be affected by measurement noise. To evaluate sensitivity beyond the nominal noise-free digital-twin benchmark, a controlled additive white Gaussian noise (AWGN) perturbation is introduced at the output of the fixed PA surrogate to characterize sensitivity to feedback-noise corruption. This experiment is intended as an algorithm-level robustness test rather than as a physical model of the complete RF feedback chain. The corrupted feedback signal is represented as $\tilde{y}(n)=y(n)+w(n)$, where $\tilde{y}(n)\in C^{L}$ denotes the noisy observation, $y(n)\in C^{L}$ is the ideal digital-PA output, and $w(n)\in C^{L}$ is the added AWGN component. The noise variance is adjusted to obtain target signal-to-noise ratio (SNR) values of 45 dB and 35 dB, representing a relatively high-SNR feedback condition and a lower-SNR perturbation condition, respectively.

**Figure 9:**
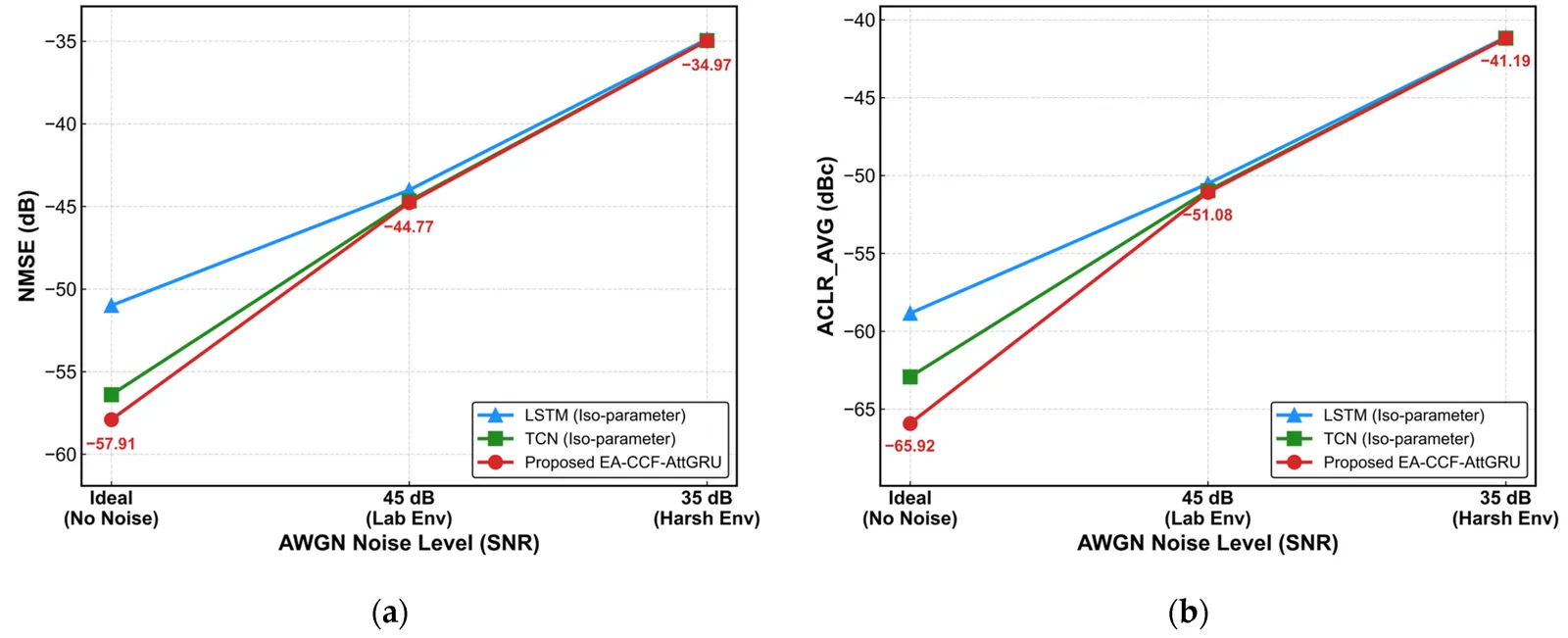
Quantitative validation of noise robustness under AWGN injection. (**a**) NMSE degradation trajectories. The proposed EA-CCF-AttGRU achieves the best ideal-state NMSE and remains competitive under 45 dB and 35 dB SNR conditions. (**b**) ACLR degradation trajectories. The proposed model provides the best ideal-state ACLR, while all evaluated models exhibit consistent degradation as the SNR decreases.

Table 6 and Figure 9 summarize the quantitative degradation of the approximately iso-parameter models under the controlled AWGN feedback-noise conditions. Under the ideal noise-free condition, the proposed EA-CCF-AttGRU achieves an ACLR of −65.92 dBc and an NMSE of −57.91 dB, compared with −62.93 dBc/−56.40 dB for the iso-parameter TCN-DPD and −58.85 dBc/−51.00 dB for the iso-parameter LSTM. At an SNR of 45 dB, the proposed model retains an ACLR of −51.08 dBc with an NMSE of −44.77 dB. When the SNR is reduced to 35 dB, the ACLR values of the evaluated models converge to approximately −41.1 dBc and the NMSE values approach −35 dB, indicating that the imposed observation-noise floor becomes the dominant limitation. These results characterize the relative sensitivity of the evaluated architectures to the controlled feedback-noise perturbation and show that the proposed EA-CCF-AttGRU remains competitive under the two investigated SNR conditions.

### 4.7. Sensitivity to Residual Fractional Timing Mismatch

Practical DPD adaptation additionally depends on synchronization between the observation and reference paths. Even after nominal delay compensation, a residual fractional timing mismatch may remain because the physical feedback path, ADC sampling, and clock synchronization cannot generally guarantee exact fractional-sample alignment. To quantify this sensitivity independently of interpolation artifacts, the fractional-delay operator used to generate the controlled offsets was first verified. A seven-tap symmetric Lagrange interpolator with tap positions {−3,−2,−1,0,1,2,3} was evaluated over the occupied 160 MHz bandwidth for $\delta /T_{s}=\{0,\pm 0.05,\pm 0.10,\pm 0.20,\pm 0.30,\pm 0.50\}$. The same interpolation coefficients were applied to the in-phase and quadrature components, and boundary samples affected by the finite interpolation support were treated identically for all conditions. The δ=0 condition reproduced the identity mapping exactly. Across the occupied bandwidth, the maximum magnitude deviation was approximately 0.0012 dB and the maximum phase error was approximately $0.022^{\circ }$. These interpolation errors are substantially smaller than the performance variations observed in the subsequent adaptation experiment and therefore do not dominate the reported sensitivity.

For the adaptation experiment, all timing conditions were initialized from the same validated clean EA-CCF-AttGRU checkpoint. Five adaptation runs were performed for each of the eleven timing offsets, giving 55 adaptation runs in total. The five seeds controlled the stochastic adaptation process, while every run started from the same clean checkpoint. All conditions used the same 150-epoch adaptation budget and the same fixed pretrained PA surrogate. The clean initialization was retained only as a reference and was not eligible for adapted-model checkpoint selection; the selected adapted checkpoint was the model with the best validation ACLR among epochs 1–150.

Defining the clean linear reference as r[n]=Gx[n], the residual relative timing offset between the cascade output and the reference was implemented by applying the corresponding opposite fractional shift to the continuous reference before temporal framing. For an ideal time-shift operator, shifting the cascade output by δ relative to an unshifted reference and shifting the reference by −δ relative to an unshifted output describe the same relative timing displacement. In the numerical implementation, this relative offset was realized using the validated seven-tap Lagrange fractional-delay operator with identical boundary treatment for all conditions. Applying the offset before temporal framing avoids introducing artificial frame-boundary discontinuities. After adaptation, every selected DPD was evaluated on the clean DPD–PA cascade with the artificial timing mismatch removed.

**Figure 10:**
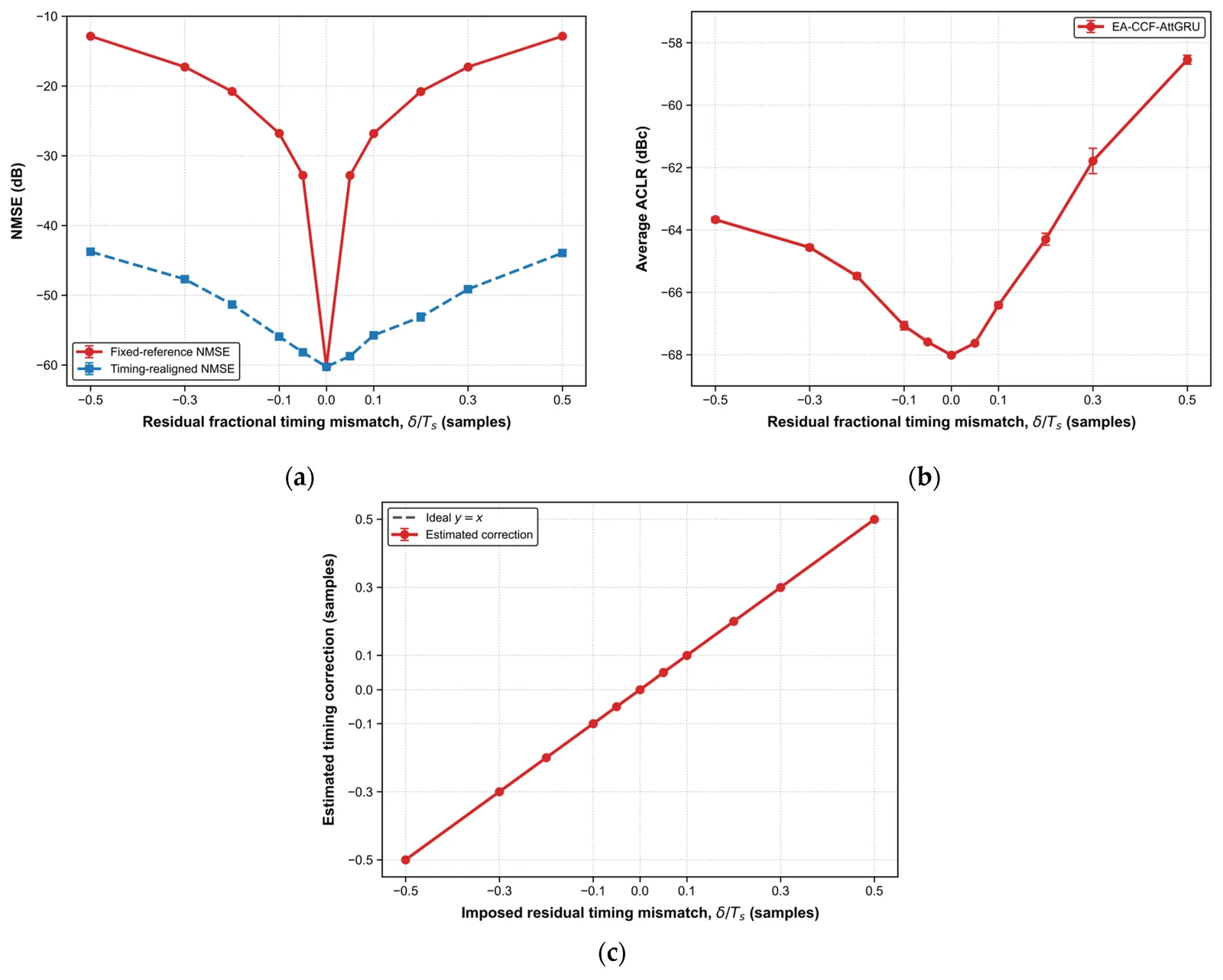
Sensitivity of the proposed EA-CCF-AttGRU to controlled residual fractional timing mismatch under the 160 MHz wideband excitation (1024-QAM, PAPR = 10.38 dB). Each point represents the mean of five adaptation runs, and the error bars denote ±1 standard deviation. (**a**) Fixed-reference NMSE and post hoc timing-realigned NMSE as functions of the residual fractional timing mismatch. The fixed-reference metric quantifies the complete output-reference mismatch, whereas the timing-realigned metric is additionally reported to separate learned timing displacement from residual waveform error. (**b**) Average ACLR as a function of residual fractional timing mismatch. (**c**) Post hoc timing correction estimated from the clean DPD–PA output; the dashed reference denotes the ideal relation y=x. Timing realignment is used only for diagnostic decomposition and is never used for training or checkpoint selection.

As shown in Figure 10, the matched δ=0 adaptation achieves a fixed-reference NMSE of approximately −60.26±0.23 dB and an average ACLR of approximately −68.01±0.06 dBc. Because the matched $\delta /T_{s}=0$ condition itself undergoes the same 150-epoch adaptation used for all timing-offset conditions in this robustness protocol, its absolute metric values need not coincide with the separately reported main benchmark in Table 4; the timing-mismatch sensitivity is therefore evaluated relative to this matched control. Because no timing correction is required in the matched condition, the timing-realigned NMSE is essentially identical. Even a small residual timing mismatch produces a pronounced penalty in the conventional fixed-reference NMSE: at $\delta =-0.05T_{s}$ and $+0.05T_{s}$, the fixed-reference NMSE degrades to approximately −32.81 dB and −32.83 dB, respectively. After post hoc removal of the dominant timing displacement, the corresponding timing-realigned NMSE values are approximately −58.19 dB and −58.73 dB, representing degradations of approximately 2.07 dB and 1.53 dB relative to the matched control. The corresponding ACLR degradations are approximately 0.42 dB and 0.38 dB, respectively.

The sensitivity generally increases with the magnitude of the residual mismatch. At $\delta =-0.10T_{s}$ and $+0.10T_{s}$, the timing-realigned NMSE degradations are approximately 4.33 dB and 4.51 dB, while the corresponding ACLR degradations are approximately 0.94 dB and 1.60 dB. At the largest investigated offsets, $\delta =-0.50T_{s}$ and $+0.50T_{s}$, the timing-realigned NMSE degradations increase to approximately 16.49 dB and 16.32 dB, respectively. The corresponding ACLR degradations are approximately 4.34 dB and 9.47 dB. The ACLR response is therefore not perfectly symmetric with respect to the sign of the residual offset. This sign-dependent difference is reported as an empirical observation and is not attributed to a specific physical mechanism in the present study.

The post hoc timing diagnostic further distinguishes learned timing displacement from residual waveform-linearization error. Across the investigated offset grid, the estimated timing correction closely follows the imposed residual mismatch, with a maximum discrepancy of approximately $4\times 10^{-4}$ samples. This result indicates that adaptation against a misaligned reference can absorb a substantial portion of the synchronization offset into the learned DPD response. Consequently, the conventional fixed-reference NMSE and EVM quantify the complete output-reference mismatch and therefore contain contributions from both residual waveform distortion and learned timing displacement. These metrics remain valid end-to-end sensitivity measures. In parallel, timing-realigned NMSE is reported as a post hoc diagnostic to estimate the residual waveform error after removal of the dominant learned timing displacement. ACLR is reported independently because a global timing shift does not impose the same direct penalty on the spectral-leakage metric. EVM was also evaluated for every timing condition and exhibited the same timing-sensitive behavior as the fixed-reference waveform-error metrics; it is therefore not duplicated as an additional panel in Figure 10. No timing realignment is used during adaptation, checkpoint selection, or the primary clean-cascade evaluation.

### 4.8. Block-Adaptive Tracking Under Dynamic Power Transitions

Unlike the fixed-parameter output-scaling evaluation in Section 4.5, this experiment examines block-wise parameter adaptation under sequential changes in operating power. The input power was varied according to the sequence 0 → −3 → −6 → −3 → 0 dB. Five block sizes, B={4096,8192,16,384,32,768,65,536}, were evaluated.

For a fair comparison, each power stage contained exactly 524,288 adaptation samples for every block size, and all block-size configurations within the same seed used the same underlying continuous input stream. Consequently, the block sizes corresponded to 128, 64, 32, 16, and 8 optimizer updates per power stage, respectively. Three independent seeds were evaluated. One optimizer update was performed after accumulation of all samples within each adaptation block, while the pretrained PA surrogate remained fixed.

**Figure 11:**
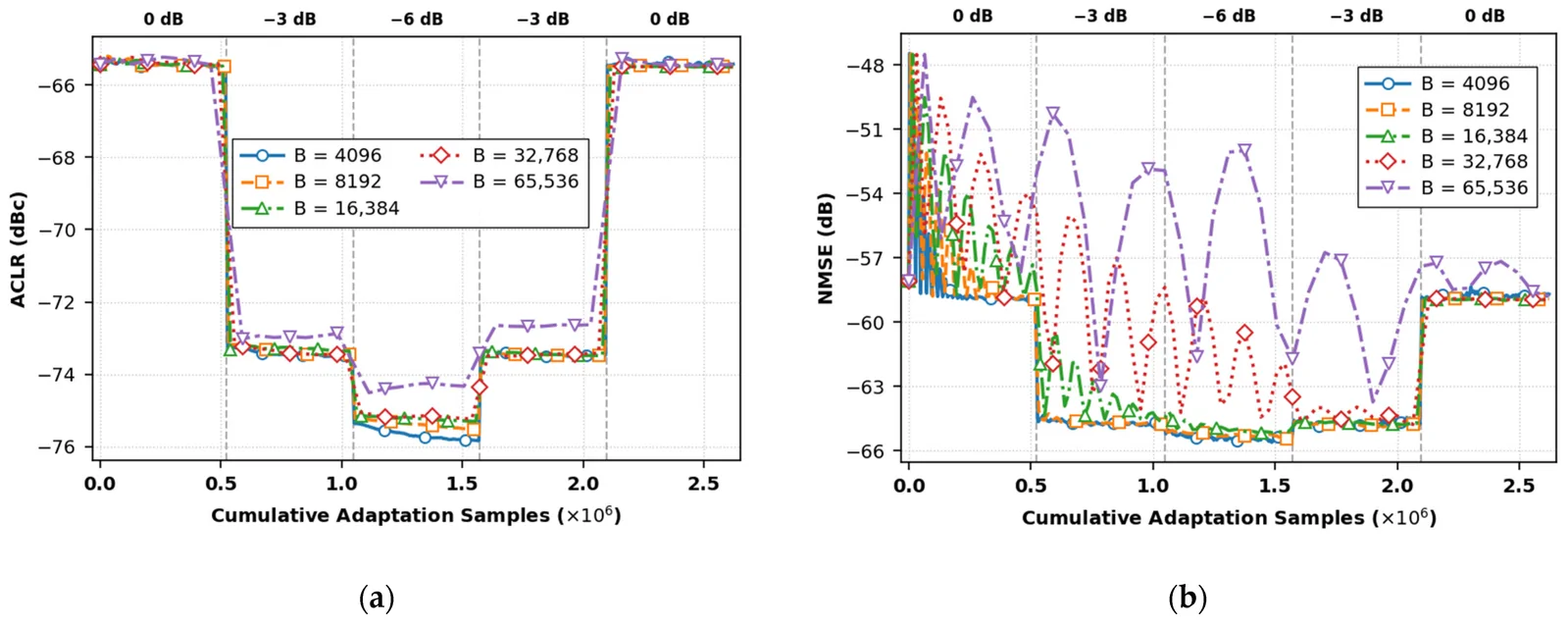
Block-adaptive tracking of EA-CCF-AttGRU under the dynamic input-power sequence 0→−3→−6→−3→0 dB. (**a**) Mean ACLR and (**b**) mean NMSE versus cumulative adaptation samples for B=4096, 8192, 16,384, 32,768, and 65,536. The curves are averaged over three seeds, and the vertical dashed lines denote power-state transitions. Every block size processes the same 524,288 adaptation samples per power stage; only the block segmentation and update frequency differ.

Steady-state ACLR for each power stage was defined as the mean of the final five post-update blocks. Convergence was declared when three consecutive blocks remained within ±0.5 dB of the corresponding steady-state ACLR, and the reported convergence sample count corresponds to the block that completed the first qualifying three-block sequence. Figure 11 shows the mean ACLR and NMSE trajectories as functions of cumulative adaptation samples. Smaller blocks provide more frequent parameter updates and therefore reach the steady-state region using fewer processed samples, whereas larger blocks reduce the number of optimizer updates required over the same input duration. The corresponding convergence, update-workload, and steady-state ACLR statistics are summarized in Table 7.

The block-size sweep reveals a direct responsiveness–update-workload trade-off. The mean convergence requirement increases from approximately 13.3k processed samples at B=4096 to 196.6k samples at B=65,536, because larger blocks produce fewer parameter updates over the same sample interval. Conversely, the accumulated software update time per power stage decreases from approximately 1.93 s to 0.35 s as B increases from 4096 to 65,536 samples. The intermediate B=16,384 configuration requires approximately 49.2k samples for convergence, 32 updates per stage, and exhibits a mean within-stage steady-state ACLR standard deviation of approximately 0.007 dB. These results demonstrate algorithm-level block-adaptive capability and quantify the trade-off between adaptation responsiveness and update workload. The measured software update times are implementation-specific PyTorch values and do not constitute FPGA/ASIC adaptation-latency measurements.

### 4.9. Post-Training Quantization Sensitivity

To assess the numerical precision sensitivity of EA-CCF-AttGRU, post-training numerical fake quantization was evaluated using four configurations: FP32, W16A16, W8A16, and W8A8, where W and A denote weight and activation precision, respectively. The trained DPD parameters were quantized using signed symmetric quantization, while intermediate activations were quantized according to their corresponding numerical ranges. Importantly, the recurrent GRU hidden state was explicitly quantized at every recurrent step. Accumulation arithmetic and the fixed PA surrogate remained in FP32 so that all DPD precision configurations were evaluated against the same PA reference. This experiment characterizes numerical precision sensitivity and parameter-storage requirements; it does not measure physical hardware power or energy consumption.

The W16A16 configuration preserves the FP32 linearization performance with only small numerical differences. Relative to FP32, its NMSE changes by −0.082 dB, the average ACLR changes by only +0.011 dB, and the EVM changes from 0.0741% to 0.0745%, while the DPD parameter storage is reduced from 23.473 to 11.736 KiB. In contrast, direct 8-bit post-training quantization causes substantial degradation. W8A16 reaches −37.24 dB NMSE and −55.20 dBc ACLR, while W8A8 further degrades to −30.06 dB NMSE and −39.48 dBc ACLR. Therefore, the trained EA-CCF-AttGRU is tolerant to the evaluated 16-bit post-training quantization but is not robust to direct uniform 8-bit post-training quantization. More aggressive 8-bit deployment would require additional methods such as quantization-aware training or architecture-aware mixed-precision allocation.

For implementation-oriented comparison, Table 8 additionally reports a normalized dense-MAC bit-cost proxy defined from the weight–activation bit-width product relative to FP32. The corresponding normalized values are 1.000, 0.250, 0.125, and 0.0625 for FP32, W16A16, W8A16, and W8A8, respectively. These values describe only the reduction in numerical bit width and are not estimates or measurements of physical energy consumption. Actual inference energy depends on device architecture, memory hierarchy, arithmetic mapping, clocking, and implementation technology and therefore requires target-specific hardware implementation.

### 4.10. Discussion: Literature Positioning, Practical Limitations, and Future Implementation

The preceding experiments evaluate the proposed architecture from complementary perspectives, including controlled model-capacity comparison, component-level ablation, capacity scaling, feedback-noise sensitivity, residual timing mismatch, block-wise adaptation, and numerical quantization. To further clarify the novelty boundary of the present work, Table 9 summarizes representative neural DPD studies that are directly related to the architectural mechanisms discussed in Section 1. The table focuses on architectural organization and implementation emphasis rather than directly comparing reported NMSE or ACLR values, because the cited studies employ different PAs, signal bandwidths, modulation formats, operating conditions, and experimental platforms.

Table 9 reinforces the novelty boundary established in the Introduction. Envelope-derived augmentation, convolutional feature extraction strategies, recurrent memory modeling, attention mechanisms, mixed-precision arithmetic, and temporal sparsity have all been demonstrated previously and are not claimed here as individually novel mechanisms. The distinction investigated in the present work is their functional organization within a unified predistorter. Specifically, the EA stage supplies established parameter-free amplitude-related features, the kernel-size-one CCF performs same-timestamp cross-channel nonlinear fusion without explicit cross-timestamp mixing, the AttGRU subsequently introduces temporal-memory evolution and adaptive recurrent-feature reweighting, and the global linear bypass provides a direct representation path for the affine compensation component. The approximately iso-parameter benchmark in Section 4.1, Section 4.2 and Section 4.3 and the ablation study in Section 4.4 provide complementary evidence that the observed performance advantage persists under comparable model capacity and that the assigned functional paths provide measurable contributions within the evaluated architecture.

This positioning should not be interpreted as a direct cross-paper performance ranking. The numerical ACLR, NMSE, EVM, parameter counts, and implementation costs reported in different studies are obtained using different PA devices, bandwidths, modulation formats, training protocols, and measurement environments. For this reason, Table 9 is restricted to architectural and implementation-oriented characteristics, whereas quantitative performance comparisons in this work are performed only under the common DPA_160MHz benchmark described in Section 4.1.

The robustness and implementation-oriented analyses also reveal several practical limitations of the present architecture. The selected EA-CCF-AttGRU configuration requires 23.473 KiB of FP32 parameter storage and maintains 32 recurrent-state values. The corresponding logical recurrent-state interface requires 32 state values to be read and 32 values to be updated per sample. These quantities characterize algorithm-level storage, state, and routing requirements rather than synthesized hardware resource utilization.

Real-time implementation for the 640-MS/s baseband sampling scenario used in this study remains implementation-dependent. A physical implementation would need to sustain at least the input sample rate, while the achievable throughput would depend on arithmetic precision, target clock frequency, resource mapping, pipelining strategy, initiation interval, and the sequential hidden-state dependency of the AttGRU. No FPGA/ASIC synthesis is performed in the present study; therefore, actual end-to-end inference latency, initiation interval, DSP/LUT/FF/BRAM utilization, power consumption, and timing closure are not claimed.

The block-adaptive experiment in Section 4.8 and the post-training quantization study in Section 4.9 provide algorithm-level evidence concerning adaptation responsiveness, update workload, and numerical precision sensitivity, but they do not establish physical hardware adaptation latency or energy consumption. In particular, the W16A16 result indicates that moderate precision reduction can preserve the evaluated linearization performance, whereas direct uniform 8-bit post-training quantization produces substantial degradation. Future work will therefore focus on target-specific FPGA/ASIC synthesis, quantization-aware and architecture-aware mixed-precision optimization, and structured pruning, followed by direct measurement of throughput, latency, resource utilization, and power consumption.

## 5. Conclusions

Wideband power amplifiers operating under high-order modulation exhibit pronounced nonlinear distortion and dynamic memory effects, motivating DPD architectures that jointly consider linearization accuracy and implementation complexity. This work developed a functionally decoupled single-box EA-CCF-AttGRU architecture in which different compensation functions are explicitly assigned to distinct structural components. Established envelope-derived features provide lightweight amplitude-related augmentation, while the kernel-size-one point-wise CCF performs instantaneous cross-channel nonlinear fusion without explicit cross-timestamp mixing. Temporal-memory evolution is subsequently modeled by the GRU, and the channel-wise attention gate provides adaptive reweighting of the recurrent representation. An independent global linear bypass further provides a direct affine representation path for the dominant linear compensation component. Accordingly, the contribution of the proposed architecture lies in this functional organization rather than in claiming envelope augmentation, recurrent modeling, attention, or residual processing as individually novel mechanisms.

Under the 160 MHz 1024-QAM evaluation condition with a PAPR of 10.38 dB, the selected 6009-parameter EA-CCF-AttGRU configuration achieves an ACLR of −65.91 dBc, an NMSE of −57.84 dB, and an EVM of 0.07%. The approximately iso-parameter comparison shows that this performance advantage persists under a trainable parameter budget comparable to those of the evaluated recurrent and temporal-convolutional baselines. The ablation and architectural-variant results further show that the envelope-assisted augmentation, point-wise CCF, recurrent-feature attention, and global linear bypass provide measurable contributions within the complete functional organization. The hardware-aware analysis characterizes 5764 dense multiplications and 5764 dense affine additions per sample, 23.473 KiB of FP32 parameter storage, and a 32-value recurrent state for the selected configuration. These results establish an observed algorithmic complexity–performance operating point rather than a hardware-optimal implementation.

Additional robustness and implementation-oriented analyses characterize the behavior of the proposed DPD beyond the primary clean-cascade benchmark. Controlled feedback-noise perturbations produce progressive performance degradation as the imposed SNR decreases, while residual fractional timing mismatch confirms the sensitivity of adaptation to feedback synchronization accuracy. The block-adaptive experiment demonstrates a trade-off between adaptation responsiveness and software update workload under dynamic power transitions. The post-training quantization study further shows that W16A16 maintains performance close to FP32 while reducing parameter storage by 50%, whereas direct uniform 8-bit quantization causes substantial degradation. These results support the relevance of the proposed architecture to contemporary wideband DPD research, in which linearization accuracy must be considered together with model capacity, adaptation behavior, numerical precision, and implementation cost.

The present study remains an algorithm-level and implementation-oriented evaluation rather than a synthesized hardware demonstration. In particular, physical throughput, end-to-end latency, initiation interval, DSP/LUT/FF/BRAM utilization, and power consumption have not been established, and the sequential hidden-state dependency of the AttGRU remains an important consideration for highly parallel implementations at high sampling rates. Future work will therefore focus on target-specific FPGA/ASIC synthesis, quantization-aware and architecture-aware mixed-precision optimization, and structured pruning, followed by direct measurement of throughput, latency, resource utilization, and power consumption.

## Figures and Tables

**Table 1:** Hardware-aware analytical complexity and software execution profile. (**a**) Per-sample operator profile of the iso-parameter DPD models. (**b**) Parameter-memory, temporal-state, and routing characteristics. (**c**) Module-wise PyTorch software execution-time profile of the proposed EA-CCF-AttGRU.

(**a**) Per-sample operator profile									
**Model**	**Parameters**	**Dense MUL**	**Dense ADD**	**Elem. MUL**	**Elem.** **ADD/SUB**	**Sigmoid**	**Tanh**	**sqrt**	**Division**
LSTM, H=37	6144	5846	5846	111	185	111	74	0	0
TCN, H=207	6003	5796	4966	4	3	0	0	1	2
Proposed EA-CCF-AttGRU, H=32	6009	5764	5764	134	165	96	32	1	0
(**b**) Parameter-memory, temporal-state, and routing characteristics									
**Model**	**FP32 Parameter Memory**		**Persistent Recurrent State**	**Recurrent State R+W/sample**	**Max. Principal Feature Width**		**Temporal Buffer**		
LSTM, H=37	24.000 KiB		74 values	148 values	37		-		
TCN, H=207	23.449 KiB		0	0	207		12,420 values		
Proposed EA-CCF-AttGRU, H=32	23.473 KiB		32 values	64 values	32		-		
(**c**) Module-wise PyTorch software execution-time profile									
**Stage**	**T = 200, Median Time (ms)**			**T = 16,384, Median Time (ms)**					
EA feature extraction	0.419680			0.337360					
1×1 CCF + LeakyReLU	0.232976			0.234448					
GRU recurrent block	0.379952			6.758400					
Attention gate	0.200704			0.251008					
Output fully connected layer + bypass + fusion	0.328704			0.218112					
Full forward	1.453936			7.336448					

Abbreviations: EA, envelope-assisted; CCF, cross-channel fusion; GRU, gated recurrent unit; LSTM, long short-term memory; TCN, temporal convolutional network; FP32, 32-bit floating-point; MUL, multiplication; ADD, addition; Elem., element-wise; R+W, read/write. The TCN temporal-buffer value is an analytical estimate for a straightforward streaming delay-line realization with kernel size 5 and dilations 1/2/4/8; it is not a measured FPGA block random-access memory (BRAM) requirement.

**Table 2:** Physical Characteristics of the Wideband Test Signal Used for DPA Behavioral Extraction.

**Parameter**	**Value**
Carriers	4 × 40 MHz
Aggregated Bandwidth	160 MHz
Baseband Sampling Rate	640 MS/s
Inter-sample Interval	1.5625 ns
Modulation Scheme	1024-QAM
Active Subcarriers	1024
IFFT Frame Size	16,384
PAPR	10.38 dB

**Table 3:** Default-capacity and approximately iso-parameter configurations of the evaluated mathematical and deep learning models. Baselines were evaluated at their default capacities and at capacities scaled to approximately 6000 trainable DPD parameters for structural comparison.

Model	Evaluation Track	Configuration/Hidden Size	Total Parameters ($N_{param}$)
GMP [3]	Analytical Baseline	Memory depth = 15	495
LSTM [32]	Default Capacity	Hidden size = 15	1172
LSTM [32]	Iso-parameter	Hidden size = 37	6144
RVTDCNN [31]	Default Capacity	Hidden channels = 15	617
RVTDCNN [31]	Iso-parameter	Hidden channels = 156	6116
TCN-DPD [24]	Default Capacity	Hidden channels = 15	435
TCN-DPD [24]	Iso-parameter	Hidden channels = 207	6003
APNRRU [28]	Default Capacity	Hidden size = 15	1393
APNRRU [28]	Iso-parameter	Hidden size = 81	6013
DeltaGRU [30]	Default Capacity	Hidden size = 15; thresholds = 0	1067
DeltaGRU [30]	Iso-parameter	Hidden size = 41; thresholds = 0	6111
TRes-DeltaGRU [30]	Default Capacity	Hidden size = 15; thresholds = 0	999
TRes-DeltaGRU [30]	Iso-parameter	Hidden size = 42; thresholds = 0	6156
Proposed EA-CCF-AttGRU	Selected Configuration	Hidden channels = 32	6009

**Table 4:** Comprehensive linearization performance metrics (ACLR, EVM, and NMSE) of the evaluated models across both default capacity and iso-parameter configurations under the 160 MHz wideband testbed (1024-QAM, PAPR = 10.38 dB).

Model	Configuration	Total Parameters ($N_{param}$)	NMSE (dB)	EVM (%)	ACLR (dBc)
GMP [3]	Memory depth = 15	495	−19.80	8.01	−44.59
RVTDCNN [31]	Default Capacity	617	−36.73	0.98	−50.41
RVTDCNN [31]	Iso-parameter	6116	−38.26	0.84	−50.87
LSTM [32]	Default Capacity	1172	−44.80	0.28	−52.74
LSTM [32]	Iso-parameter	6144	−51.00	0.15	−58.85
TCN-DPD [24]	Default Capacity	435	−48.55	0.20	−54.95
TCN-DPD [24]	Iso-parameter	6003	−56.40	0.10	−62.93
APNRRU [28]	Default Capacity	1393	−45.82	0.30	−54.83
APNRRU [28]	Iso-parameter	6013	−45.78	0.26	−53.75
DeltaGRU [30]	Default Capacity	1067	−48.72	0.20	−57.77
DeltaGRU [30]	Iso-parameter	6111	−56.14	0.09	−63.94
TRes-DeltaGRU [30]	Default Capacity	999	−51.81	0.15	−59.33
TRes-DeltaGRU [30]	Iso-parameter	6156	−56.97	0.09	−64.51
Proposed EA-CCF-AttGRU	Selected Configuration	6009	−57.84	0.07	−65.91

The APNRRU, DeltaGRU, and TRes-DeltaGRU results, including both their default-capacity and iso-parameter configurations, are single-run results obtained using the same dataset split and training/evaluation pipeline; they are not included in the five-seed statistics of the original primary comparison.

**Table 5:** Ablation and architectural-variant analysis under 160 MHz excitation. All variants use the same base recurrent hidden dimension of H=32. (**a**) Linearization performance. (**b**) Architecture-level arithmetic, nonlinear-operation, and parameter-memory costs. (**c**) Relative performance–cost trade-offs with respect to the full EA-CCF-AttGRU configuration.

(**a**) Linearization performance										
**Model Variant**	**Architectural Modification**			**Parameters**		**EVM (** **%** **)**	**NMSE (dB)**		**ACLR (dBc)**	
Proposed	EA-CCF-AttGRU (1×1 CCF only)			6009		0.074	−57.84		−65.91	
Model A	without EA feature augmentation			5977		0.090	−55.40		−63.27	
Model B	without point-wise CCF; EA features directly fed to the GRU			4777		0.073	−56.10		−64.95	
Model C	without Attention Gate			4953		0.091	−55.04		−63.16	
Model D	without Global Linear Bypass and Adaptive Residual Fusion			6002		0.088	−54.99		−63.14	
Model E	with Dual-Track Convolution (1×1 + Dilated)			6217		0.076	−56.89		−64.31	
(**b**) Architecture-level hardware-aware cost										
**Variant**	**Params**	**Dense MAC/sample**	**Dense MUL/ADD**	**Elem. MUL**	**Elem. ADD/SUB**	**Sigmoid**	**Tanh**	**LeakyReLU**	**sqrt**	**FP32** **Memory (KiB)**
Full	6009	5764	5764/5764	134	165	96	32	16	1	23.473
w/o EA	5977	5732	5732/5732	130	162	96	32	16	0	23.348
w/o CCF	4777	4548	4548/4548	134	165	96	32	0	1	18.660
w/o Attention	4953	4740	4740/4740	102	165	64	32	16	1	19.348
w/o Bypass/Fusion	6002	5760	5760/5760	132	163	96	32	16	1	23.445
Dual-track	6217	5956	5956/5956	134	181	96	32	16	1	24.285
(**c**) Relative performance–cost trade-off										
**Variant**	**Dense MAC Change vs. Full (%)**		**FP32 Parameter-Memory Change vs. Full (%)**			**ACLR Degradation vs** **.** **Full (dB)**				
Full	0.00		0.00			0.00				
w/o EA	−0.56		−0.53			+2.64				
w/o CCF	−21.10		−20.50			+0.96				
w/o Attention	−17.77		−17.57			+2.75				
w/o Bypass/Fusion	−0.07		−0.12			+2.77				
Dual-track	+3.33		+3.46			+1.60				

The relative dense-MAC and FP32 parameter-memory changes in (c) are calculated with respect to the full EA-CCF-AttGRU configuration. Negative values denote cost reductions, whereas positive values denote cost increases. ACLR degradation is defined as the difference between the variant ACLR and the full-model ACLR; therefore, a positive value denotes degraded linearization performance. These quantities are architecture-level analytical indicators and should not be interpreted as synthesized DSP, LUT, FF, BRAM, or logic-gate savings. Dense MAC counts are reported together with the corresponding dense multiplication/addition counts under the adopted analytical counting convention. Nonlinear and special-function evaluations are reported separately and are not converted into equivalent MACs. These values are architecture-level analytical counts and do not represent synthesized DSP, LUT, FF, or BRAM utilization.

**Table 6:** Performance degradation with different AWGN impairment levels. All evaluated models are within the iso-parameter range (approximately 6000 parameters).

Model	Ideal NMSE (dB)	Ideal ACLR (dBc)	45 dB NMSE (dB)	45 dB ACLR (dBc)	35 dB NMSE (dB)	35 dB ACLR (dBc)
LSTM (iso-parameter)	−51.00	−58.85	−44.01	−50.53	−34.88	−41.13
TCN-DPD (iso-parameter)	−56.40	−62.93	−44.67	−50.97	−34.96	−41.18
Proposed EA-CCF-AttGRU	−57.91	−65.92	−44.77	−51.08	−34.97	−41.19

The robustness results were recomputed using one fixed checkpoint for each model under all three noise conditions. The ideal/no-noise values therefore correspond to the same checkpoints used for AWGN injection and may differ slightly from the five-seed mean values reported in Table 4.

**Table 7:** Block-adaptive responsiveness and software update-workload trade-off under dynamic power transitions. Convergence statistics are averaged over the four power transitions and three seeds.

Adaptation Block Size, B (Samples)	Convergence Samples, Mean ± SD	Updates per Power Stage	Mean Steady-State ACLR SD (dB)	Measured PyTorch Update Time per Stage (ms)	−3 dB ACLR	−6 dB ACLR	Return −3 dB ACLR	Return 0 dB ACLR
4096	13,312 ± 3547	128	0.0142	1926.4	−73.484	−75.838	−73.462	−65.423
8192	24,576 ± 0	64	0.0094	987.9	−73.466	−75.533	−73.469	−65.473
16,384	49,152 ± 0	32	0.0073	683.6	−73.365	−75.297	−73.482	−65.502
32,768	98,304 ± 0	16	0.0107	405.5	−73.462	−75.218	−73.427	−65.484
65,536	196,608 ± 0	8	0.0347	350.4	−72.919	−74.295	−72.657	−65.451

The reported update time is PyTorch software execution time on the test platform and is not interpreted as hardware adaptation latency.

**Table 8:** Post-training 8/16-bit quantization sensitivity of EA-CCF-AttGRU on the DPA_160MHz test set.

Precision	W/A (bit)	NMSE (dB)	ACLR (dBc)	EVM (%)	Parameter Storage (KiB)	Storage Reduction	Hidden-State Storage (bytes)	Normalized Dense-MAC Bit-Cost Proxy
FP32	32/32	−57.8445	−65.9052	0.0741	23.473	0%	128	1.000
W16A16	16/16	−57.9267	−65.8939	0.0745	11.736	50%	64	0.250
W8A16	8/16	−37.2352	−55.1962	0.4252	5.868	75%	64	0.125
W8A8	8/8	−30.0602	−39.4779	1.1462	5.868	75%	32	0.0625

**Table 9:** Architectural positioning of representative neural DPD approaches.

Method	Key Architectural Characteristic	Relation to the Present Work
ARVTDNN [5]	Envelope-assisted feature augmentation for nonlinear PA representation	Demonstrates the effectiveness of amplitude-related feature enhancement
LUT-assisted BiLSTM [16]	Cascaded static LUT compensation and recurrent neural modeling	Uses separated static and dynamic compensation paths rather than functional integration within one neural architecture
GRU-/LSTM-Attention DPD [8,10]	Recurrent memory modeling with attention-based feature weighting	Establishes attention-assisted recurrent DPD; the present work investigates its integration with explicitly separated feature construction and temporal-memory pathways
TCN-DPD [24]	Dilated temporal convolution for memory-effect modeling	Represents convolution-based temporal feature extraction without explicit recurrent state evolution
MP-DPD/ DeltaDPD [29,30]	Complexity-oriented optimization through reduced numerical precision or temporal sparsity	Focuses on numerical or execution efficiency rather than assigning explicit functional roles to different architectural pathways
EA-CCF-AttGRU (this work)	Envelope augmentation, point-wise cross-channel fusion, AttGRU temporal modeling, and linear bypass combined in a unified architecture	Investigates functional role assignment among feature construction, nonlinear fusion, temporal memory, adaptive weighting, and linear compensation paths

## Data Availability

The OpenDPD dataset analyzed in this study is publicly available. It can be accessed at https://github.com/lab-emi/OpenDPD (accessed on 15 July 2026).
